# Reinforcing Restorative Dentistry: The Dual Role of Carbon Nanotubes in Material Enhancement and Therapy

**DOI:** 10.1155/ijod/5535891

**Published:** 2025-10-28

**Authors:** Ranjeet A. Bapat, Sumit V. Bedia, Aarti S. Bedia, Esther Kok Sook Kuan, Abhishek Parolia, Anshad Mohamed Abdulla, Prachi R. Bapat, Tanay V. Chaubal, Amirhossein Sahebkar, Waleed H. Almalki, Prashant Kesharwani

**Affiliations:** ^1^Division of Restorative Dentistry, School of Dentistry, IMU University, Kuala Lumpur 57000, Malaysia; ^2^Bharati Vidyapeeth (Deemed to be University) Dental College and Hospital, Navi Mumbai 400614, Maharashtra, India; ^3^Klinik Pergigian, Temerloh Ministry of Health, Temerloh, Malaysia; ^4^Department of Endodontics, College of Dentistry and Dental Clinics, University of Iowa, Iowa, USA; ^5^Department of Pediatric Dentistry and Orthodontic Sciences, King Khalid University, Abha, Saudi Arabia; ^6^Borivali (West), Mumbai 400091, Maharashtra, India; ^7^OU College of Dentistry, Oklahoma City 73117, USA; ^8^Center for Global Health Research, Saveetha Medical College and Hospitals, Saveetha Institute of Medical and Technical Sciences, Saveetha University, Chennai, India; ^9^Biotechnology Research Center, Pharmaceutical Technology Institute, Mashhad University of Medical Sciences, Mashhad, Iran; ^10^Department of Pharmacology and Toxicology, Faculty of Pharmacy, Umm Al-Qura University, Makkah, Saudi Arabia; ^11^Department of Pharmaceutical Sciences, Dr. Harisingh Gour Vishwavidyalaya (A Central University), Sagar 470003, Madhya Pradesh, India; ^12^University Institute of Pharma Sciences, Chandigarh University, Mohali, Punjab, India

**Keywords:** antimicrobial properties, biocompatibility, carbon nanotube, nanomaterials, restorative dentistry

## Abstract

Carbon nanotubes (CNTs) have garnered considerable interest in dentistry due to their exceptional properties, including high tensile strength (TS), low weight, chemical stability, and antimicrobial activity. These attributes make CNTs valuable for enhancing the mechanical performance and therapeutic potential of dental materials. Recent advances have demonstrated their effectiveness in dental composites, improving flexural strength (FS), wear resistance, and minimizing polymerization shrinkage. Furthermore, their antimicrobial properties contribute to the prevention of secondary caries and increase the longevity of restorations. Beyond restorative uses, CNTs show promise in drug delivery and tissue engineering, particularly in bone regeneration and periodontal therapy. However, concerns about biocompatibility remain, primarily due to potential cytotoxicity associated with metal impurities. Surface functionalization is critical in improving biological interactions and reducing toxicity. Despite these challenges, CNT–based dental materials represent a promising frontier in strengthening restoration and combating microbial threats. This review explores the dual role of CNTs in restorative dentistry and targeted therapeutics, synthesizing current evidence and outlining future directions for clinical application.

## 1. Introduction

Nanotechnology involves the study and technology of functional structures at the nanoscale, ranging from 1–100 nm [1–7]. It is used in various fields to harness unique characteristics such as enhanced conductivity, stability, magnetic properties, or color-changing properties. A greater surface area of nanomaterials allows them to be applied in areas like biocatalysis, drug delivery, and energy solutions [8–14]. The positive charge and increased surface area in nanomaterials enhance the antibacterial property [15–19]. It produces superior materials or enhances current ones, establishing the foundation for innovative techniques in disease diagnosis and prevention [20].

Nanodentistry, which has emerged in the 21st century, utilizes nanotechnology for diagnosis, treatment, and restorative care, providing instruments such as nanorobotics and nanomaterials [21, 22].

In the aspect of dentistry, nanomaterials are incorporated in different fields such as imaging, diagnostics, therapeutic agents, endodontics, preventive oral care, and advanced nanocomposites [23–31]. Besides, it is also used in the development of growth factors, scaffolds, and biomodulation techniques for tissue repair in dentistry. The extensive array of dental nanomaterials has increased overtime, with numerous variants investigated for industrial and scientific applications.

There are different types of nanomaterials used in dentistry, such as carbon nanotubes (CNTs), graphene, and graphene quantum dots (GQDs). CNTs are cylindrical structures which are made-up of rolled-up graphite sheets [32, 33]. There are two main types of CNTs based on the number of graphene layers: (i) single-walled CNTs (SWCNTs) consist of a single graphene layer which are arranged in hexagonal bundles and (ii) multiwalled CNTs (MWCNTs) are multiple graphene layers arranged concentrically [34, 35]. The SWCNTs consists of one layer of graphene (diameter range: 0.4–2 nm), whereas the MWCNTs have many layers of graphene (outer diameter range: 2–100 nm and inner diameter range: 1–3 nm). Due to their high aspect ratio, CNTs are well-suited for biological applications [36].

Application of CNTs is vast in drug delivery, due to their outstanding physical and chemical properties, such as excellent tensile strength (TS), lightweight, unique electronic structures, and chemical and thermal stability [37, 38].

Recent advancements have demonstrated that the incorporation of CNTs into dental composites can significantly improve mechanical properties such as flexural strength (FS), wear resistance, and reduced polymerization shrinkage [39]. The advancement of new and efficient drug delivery systems utilizing CNTs can enhance the pharmacological characteristics of various medications [40].

Moreover, the antimicrobial properties of CNTs offer promising avenues for preventing secondary caries and enhancing the longevity of dental restorations [41]. This is particularly relevant given the increasing prevalence of antibiotic resistance and the need for innovative solutions to combat microbial challenges in oral health. Despite the promising applications, concerns regarding the biocompatibility and safety of CNTs remain paramount. Research indicates that factors such as the size, surface functionalization, and purity of CNTs play critical roles in their interaction with biological systems. While some studies advocate for their biocompatibility, others highlight potential cytotoxic effects associated with impurities often found in commercially available CNTs [42]. Therefore, a comprehensive understanding of these materials' physicochemical properties is essential to harness their full potential while ensuring patient safety. This study aims to provide a recent update on the utilization of CNTs in enhancing the properties of dental restorative and resin-based materials, exploring their mechanisms of action, applications in drug delivery systems, and implications for future dental practices. By synthesizing current research findings, we seek to elucidate the dual role of CNTs as both innovative materials for restoration and as vehicles for targeted therapeutic delivery within the oral cavity for treatment of periodontal disease, cancer, endodontic infection, bone regeneration around implants, and tissue engineering. This review highlights both the potential benefits and challenges associated with using CNTs in dentistry.

## 2. Classification of Nanomaterials

Nanomaterials can be classified into three main categories based on their composition:

### 2.1. Biodegradable Nanomaterials

• Also known as organic nanomaterials, typically include liposomes, dendrimers, ferritin, micelles, and other materials, that are both recyclable and safe for consumption.
• Nanocapsules, which have a porous core, are sensitive thermally and to electromagnetic radiation.
• They are widely used in medicine and dentistry for delivering drugs to specific targets.

### 2.2. Biologically Inert Nanomaterials

• Also known as inorganic nanomaterials that are based on metal and metal oxides are often categorized in this group.
• Metal nanomaterials have unique optoelectrical characteristics, are made solely of metal precursors, and have localized surface plasmon resonance (LSPR) characteristics.
• Metal oxide nanomaterials such as zinc oxide (ZnO), cerium oxide, titanium oxide (TiO_2_), aluminum oxide, silicon dioxide (SiO_2_), and iron oxide are commonly produced oxides that exhibit remarkable characteristics in comparison to other metallic counterparts.
• Ceramic nanomaterials are produced by subjecting inorganic nonmetallic solids to cooling and heating processes. These materials may exist in various forms such as crystalline structures, heavy, permeable, or cylindrical structures. They are used in photodegradation, catalytic activity, and imaging.
• Semiconductor nanomaterials have characteristics that lie between those of metals and nonmetals. By adjusting their spectrum gaps, their characteristics may be greatly modified, rendering them highly valuable in the fields of photocatalysis, photo optics, and electronic devices.

### 2.3. Carbon-Based Nanomaterial

• Nanomaterials derived from carbon include a wide variety of compounds, including graphene, fullerenes, CNTs, nanofibers, carbon black, and, on rare occasions, activated carbon.
• Fullerenes are carbon particles that have a circular shape. They are composed of carbon atoms that are bound together through sp^2^ hybridization. The size of these molecules can vary, ranging from 8.2 nm for each layer to 36 nm for several layers.
• Graphene is a flat sheet of carbon particles organized in a hexagonal structure. It is two-dimensional and has a width of around 1 nm.
• CNTs are cylindrical structures made from interwoven graphene nanofoils. They can be as thin as 0.7 nm for single-layered tubes and up to 100 nm for multilayered tubes. The length of these tubes can range from a few micrometers to millimeters.
• Carbon nanofibers are carbon structures that have a cup- or cone-shaped twist, unlike cylindrical graphene nanofoils.
• Carbon black is a formless carbon material that is typically spherical in shape and has a diameter between 20 and 70 nm.

## 3. Need for CNTs as a Biomaterial in Dentistry

CNTs have attracted considerable attention in dentistry due to their distinctive characteristics and potential applications. CNTs offer high strength and durability for dental materials, enhancing fracture toughness and reducing cracks in restorations. They also have antimicrobial properties, preventing bacterial colonization and biofilm formation. Their unique structure improves bonding, enhancing restoration longevity. Additionally, their electrical conductivity makes them ideal for smart dental materials. While other nanoparticles (NPs) like silica, titanium dioxide, or silver NPs (AgNPs) have their own unique advantages, the combination of mechanical strength, electrical and thermal conductivity, antibacterial properties, and versatility in functionalization makes CNTs particularly beneficial for advanced applications in dentistry [43, 44].

## 4. Structure of CNTs

**Figure 1:**
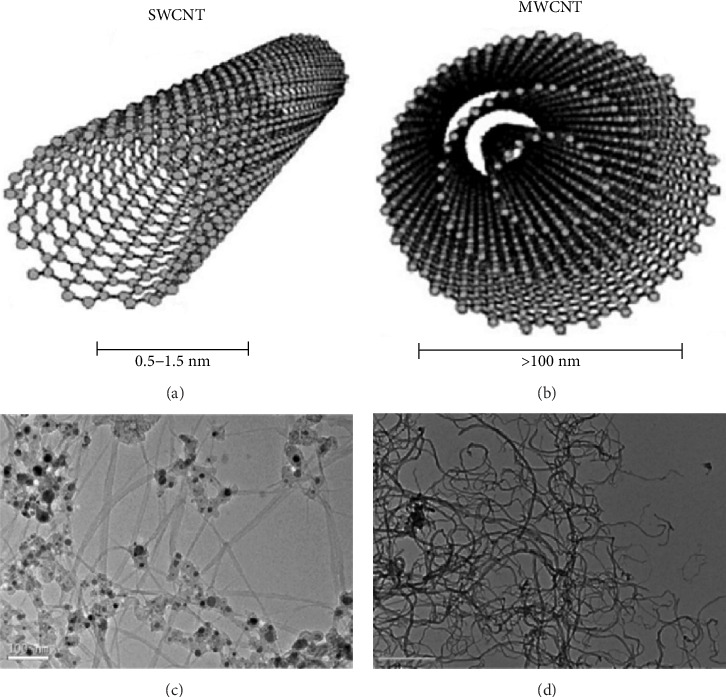
Pictorial graph of (a) single-walled carbon nanotube and (b) multiwalled carbon nanotube and SEM image of (c) MWCNT and (d) SWCNT (reprint with permission from Ali et al. [45]).

Structurally, CNTs consist of rolled graphene sheets that represent the carbonaceous nanomaterial in them [45]. These graphene sheets contain a hollow and cylindrical form featuring a hexagonal arrangement of hybridized columnar (barrel-shaped) atoms [41, 46]. The two most common forms of CNTs as shown in Figure 1 are: SWCNTs and MWCNTs.

The SWCNTs comprise of a single graphene sheet, while MWCNTs are fabricated by rolling multiple graphene sheets into one tube possessing multiple nested cylinders of differing diameters. The diameter of SWCNTs could range between 0.4 and 2 nm and that of MWCNTs can range between 2 and 100 nm [41]. Based on the method of synthesis, CNTs can extend to several millimeters of length. The graphene sheets rolled at different angles produce CNTs in three different configurations: the achiral chair type (two sides of the hexagon are oriented perpendicular to the CNT's axis), the achiral zigzag type (two sides of the hexagon are parallel to the CNT's axis), and the chiral type (any pair of hexagon sides are positioned at an angle other than 0 or 90° to the CNT's axis) [41]. These differences in synthetic processes unfortunately do not negate the future probability of defects like the Stone–Wales defect that could appear in CNTs. Due to their structural differences, MWCNTs are more equipped to strengthen polymer composites. Recently, helical MWCNTs, which are a different type of MWCNTs, are preferred and used more frequently due to their better physical properties [47, 48].

## 5. Fabrication Techniques of CNTs

**Figure 2:**
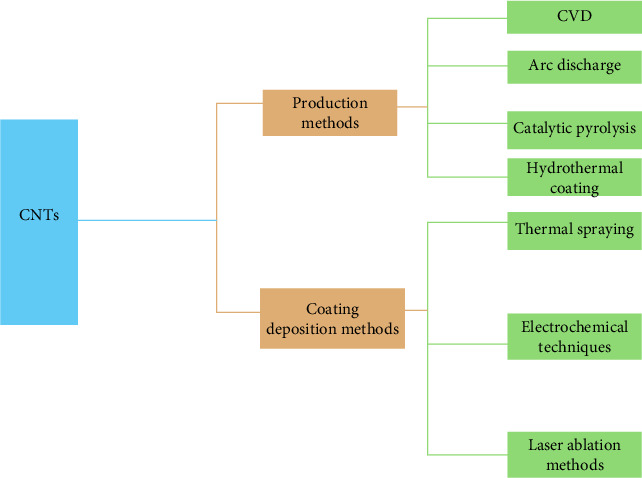
Different techniques of CNT synthesis.

Different synthesis techniques for CNTs are shown in Figure 2, include the arc-discharge method, the sol–gel process, laser ablation method, chemical vapor deposition (CVD), vapor-phase growth, flame synthesis method, electric arc discharge, and plasma-assisted growth [49, 50]. CVD technique is routinely used for large scale production of CNTs. In this method, the hydrocarbons are employed as precursors in the presence of metallic catalysts at temperatures ranging from 500 to 1000°C. Decomposition of hydrocarbons occurs at this temperature and as this temperature cools down, eventual development of CNTs occurs. CVD technique produces little impurities and consumes less energy in comparison to laser ablation and arc discharge. Arc discharge technique is centered on a high voltage arc to produce plasma. Here, direct current is passed through graphite electrodes beneath low pressure inert argon. Arc discharge technique produces a high quality of CNTs in comparison to CVD technique. In laser ablation, the furnace utilizes a pulsed laser as a heat source to dispense heat. In this method, graphite releases a high carbon vapor, and carbon vapor along with argon are moved utilizing the flowing gas. The advantage of the laser method is that they produce high quality CNTs in a large amount [51, 52]. These fabrication techniques markedly affect the structural integrity and mechanical properties of CNTs employed in restorative dentistry. CVD yields CNTs with regulated dimensions and little impurities, rendering them appropriate for uniform distribution in dental resins, therefore, improving FS and wear resistance [53]. Conversely, arc discharge and laser ablation produce CNTs characterized by enhanced crystallinity and decreased structural flaws, resulting in improved mechanical reinforcing capabilities in composite materials [41]. Nonetheless, these techniques frequently necessitate postsynthesis purification to diminish metallic contaminants that could impact biocompatibility. The synthesis pathway influences surface functionalization capabilities, which are essential for enhancing resin compatibility and reducing cytotoxicity in dental applications [46].

## 6. Properties and Characteristics of CNTs

Carbon nanostructures have exhibited phenomenal material properties like superior strength, mechanical stiffness, and elasticity as well as an advanced level of electrical and thermal conductivity [54, 55]. They are also said to have excellent antibacterial properties against a wide spectrum of bacterial species like *Streptococcus mutans*, *Staphylococcus aureus*, *Escherichia coli* (*E. coli*), *Micrococcus lysodeikticus*, *Salmonella typhimurium*, and *Bacillus subtilis*. Various research have revealed that the antibacterial properties of CNTs by means of physical contact and collisions, thereby causing the puncture of the bacterial cell membrane and eventually damaging them [49, 56, 57]. Due to these exceptional characteristics of CNTs, there is significant inquisitiveness regarding their application in numerous fields including dentistry. Therefore, it is vital to assess the biocompatibility and safety of CNTs concerning human health. Research indicates a lack of consensus among researchers regarding the safety of use of CNTs [49].

Most CNTs contain notable amounts of impure metals like cobalt, nickel, iron, and platinum which can cause genotoxic and cytotoxic effects [58]. The toxicity is mainly due to its fabrication methods. Metal residues are the leftover from the fabrication process [59]. Also, there are various other factors that instigate cytotoxicity like surface area, length and size distribution, the type of cell, cellular uptake, coating, et cetera [60], that could interact with each other, resulting in contradictory findings in the literature. As a result, some researchers have reported about the toxicity of CNTs to humans and can damage mammalian cells (mostly from researchers with environmental concerns) [61, 62]; others have advocated that they are biocompatible [63].

Research on CNT integrated materials in cellular tests, highlighting their applicability, biocompatibility, and functional efficacy are provided in Table 1. It shows that when integrated into polymer matrices like polymethyl methacrylate (PMMA), polycaprolactone (PCL), PHBV, and PLGA, these materials show improved mechanical properties and biological reactions without significant cytotoxicity. The table also highlights the potential of CNT-incorporated materials in biomedical applications, but requires careful concentration optimization to balance usefulness and biosafety. CNTS are revolutionizing dental materials, offering enhanced mechanical strength, fracture toughness, and an elastic modulus. These materials offer clinical advantages like increased resistance to occlusal forces, prolonged durability, and less restorative failure. CNTs also offer thermal conductivity and electrical conductivity for biosensing applications. These developments make CNTs a promising candidate for next-generation dental restorations and the comparative details are provided in Table 2.

## 7. Mechanism of Action of CNTs

### 7.1. Antimicrobial Action of CNT

**Figure 3:**
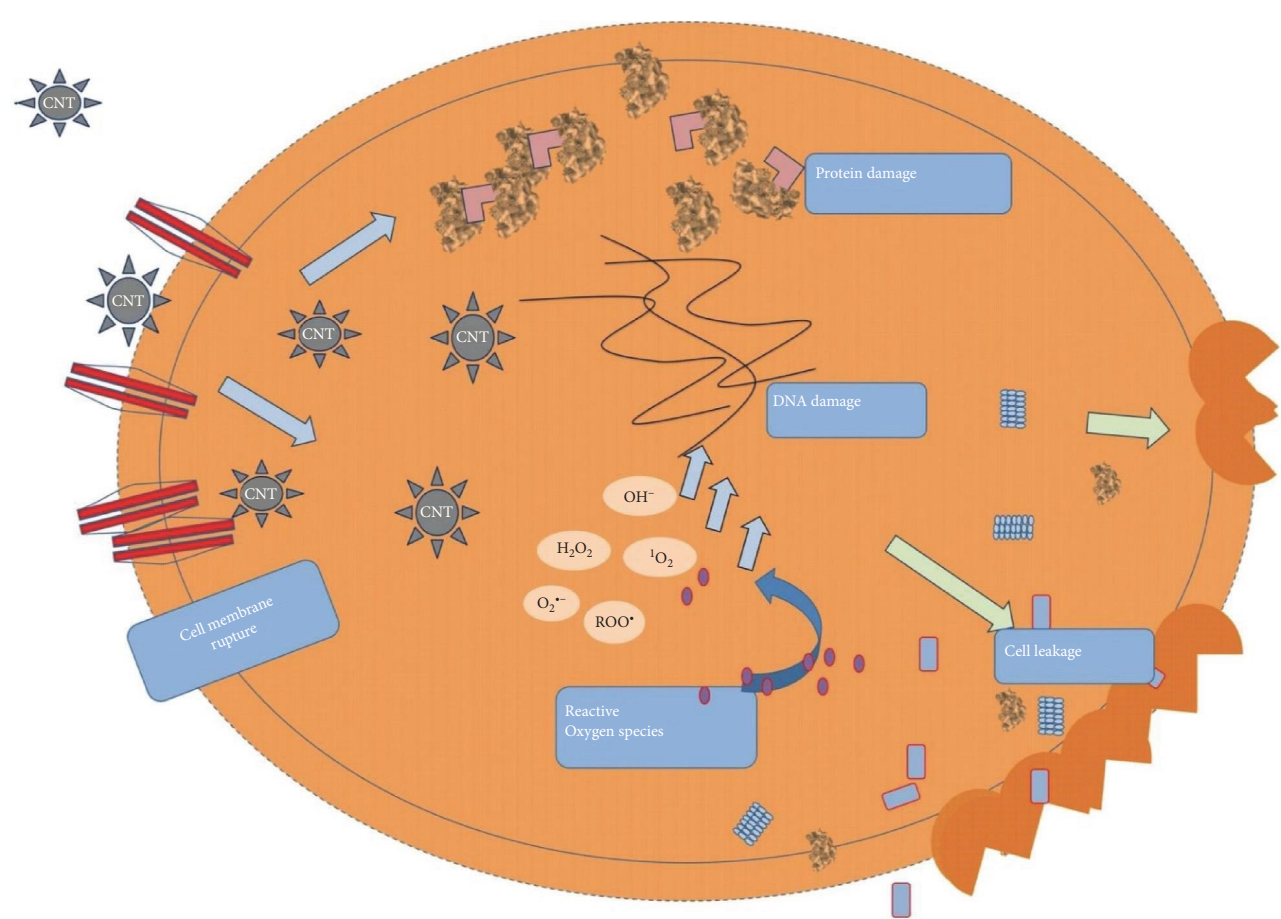
Mechanism of action of CNT.

The antimicrobial characteristics of CNTs primarily depend on factors such as their structure, surface alteration, particular organism, and their immediate surroundings. The antimicrobial actions of CNTs primarily involve infiltrating the cell wall of microorganisms and causing physical disruption. Oxidative stress is caused by the generation of harmful substances and reactive oxygen species (ROS), which strip electrons from the surface of microorganisms and lead to cell death [75]. Several researchers have verified that reducing CNT sizes enhances surface-to-volume ratio, causing more robust attachment with the microbial cell membranes of microorganisms. Cell membrane disruptions, alteration of metabolic processes, structural changes, enhanced efflux of plasmid DNA, RNA, and cytoplasmic components are the major bacteriostatic modes of action of CNTs [76–78], as shown in Figure 3. This depicts the possible mechanisms by which CNTs may induce damage to cells. They can cause DNA damage and produce ROS including nitrogen dioxide (NO_2_) and peroxy radicals (ROO^•^), resulting in oxidative stress. Furthermore, CNTs may compromise cell membrane integrity, leading to cellular leakage and rupture. The cumulative effects of oxidative stress, DNA damage, and membrane rupture indicate that CNTs may induce cytotoxicity, contingent upon variables such as functionalization, dosage, and time of exposure.

CNTs as innovative carriers for antimicrobial agents will enhance their bioavailability and streamline targeted therapy. In 2007, Kang et al. [79] initially reported the size-dependent antibacterial activities of SWCNTs against *E. coli*. According to their supplementary research, SWCNTs exhibit more toxicity towards microorganisms, as well as gram-negative and gram-positive bacteria, as compared to MWCNTs [79–81]. CNTs with a smaller diameter would have improved ability to penetrate the cell wall.

CNTs' antibacterial effectiveness may vary based on microbe type, shape, cell surface mechanical properties, and growth state [76, 82, 83]. Chen et al. [84] propose nano-darts as the main cause of bacteria death [82]. Gram-positive bacteria are particularly vulnerable to SWCNTs due to their spherical anatomy and fragility of membrane [77, 84]. Free-floating and cylindrical cells along with biofilms are resistant to CNTs [85]. Antibiotics and antimicrobials are delivered by chemically stable CNTs. Combating antibacterial resistance with antibiotic-loaded CNTs is promising. Due to their intrinsic antibacterial action, drug-resistant pathogens have increased the importance of CNTs research. The research findings on antimicrobial properties of CNTs-incorporated materials that contribute to the prevention of secondary caries properties and increase the longevity of restorations are provided in Table 3.

MWCNT nanofluids as biomaterials with common antibiotics like streptomycin and kanamycin, have superior properties in treating *M. fortuitum*, including higher penetration into the bacterial membrane, increased efficiency in lower concentrations, and lower bacterial resistance to antibiotics [77]. Treatment of the resistant *Klebsiella pneumoniae* strain with +f-MCWNTs antibiotic proves antimicrobial efficacy in lower dosages, reduces antibiotic resistance, and increases cell wall permeability to the antibiotic [90]. They would be novel medical device and prosthetic implant production alternatives [91–93]. Malek et al. [93] found that silicone materials coated with well oriented MWCNTs can prevent biofilm formation by 60% and may be used in medical device manufacture. CNTs have potential in biomedicine, but their hydrophobic nature and toxicity can disadvantage them. Functionalized CNTs (f-CNTs) are chemically modified to introduce specific functional groups or molecules [94]. Functionalization methods for f-CNTs include covalent attachment of functional groups and noncovalent adsorption or wrapping of molecules, determining their specific application and desired properties [95].

Polar or charged groups improve CNT solubility, dispersibility, and incorporation into polymers, ceramics, and metals. Functional groups enhance load transfer, composite mechanical characteristics, biomedical delivery, and antibacterial properties, enhancing contact with cellular membranes [76]. MWCNTs with amine, carboxyl, nitrogen ions, and ethanolamine exhibit potent antibacterial properties against *E. coli* and *S. aureus* when used in medical devices [96–98]. Research shows that amine-functionalized MWCNTs (f-MWCNTs) significantly increase the minimum inhibitory concentration (MIC) of *E. coli* and *S. aureus* compared to PCL [96].

## 8. Dental Applications of CNTs as a Novel Biomaterial

CNTs have been used as a novel biomaterial in dentistry. The characteristics of the research findings are presented in Table 4. Following are the applications of CNT in various fields of dentistry.

### 8.1. Restorative Biomaterials

#### 8.1.1. Composite Resins

Nanotechnology has been progressively utilized in many domains of dentistry, especially for the enhancement of materials. Integrating NPs into dental composites is essential for enhancing their mechanical properties, including fracture toughness, FS, TS, and wear resistance, hence, prolonging the durability of restorations [148]. Dental composites contain hydrophobic resin and inert filler. Light or activators cause polymerization and resin cure. Cure composites have good wear resistance, flexural, and compressive strength [149]. Their considerable shrinkage (<4%) can disadvantage them compared to microfractures [150–152]. Fillers including silica, quartz, and silica glass reduce polymerization shrinkage and improve composite mechanical properties. Additions of CNTs have been incorporated in numerous research studies to assess their effectiveness. Zhang et al. [99] developed a sandwich-like structure to enhance the use of SWCNTs in dental resin-based composites (DRCs). A thin layer of nano-SiO_2_ was deposited onto the oxidized surface of SWCNTs and a layer of allyl-terminated was added to the nano-SiO_2_ to achieve an allyl-terminated silane layer on the outside, a nano-SiO_2_ layer in the middle and an amino-modified silane layer inside. Various properties of this surface modified SWCNTs were studied using transmission electron microscopy (TEM), Fourier transform infrared spectroscopy (FTIR), and MTS Synergie 100 testing machine. It was observed that inclusion of the modified SWCNTs into the dental-based resin composites improves its FS and other mechanical properties [99].

Zheng et al. [100] evaluated the microstructure, strength, and fracture toughness of experimental yttria partially stabilized zirconia, In-Ceram zirconia slip, degree of conversion (DC) Zirkon, and In-Ceram dry pressed. He discovered that in IZ, the mechanism of toughening could be attributed to several factors: contact shielding, crack deflection due to alumina grains, microcrack nucleation, and phase transformation due to zirconia particles. In YZ (Y_2_O_3_ TZP) and DZ, the differences in fracture toughness and strength were linked to variations in the grain size, amount of stabilizing oxide (yttria), processing method used and their influence on the porosity and metastability of tetragonal grains. Philipp et al. [153] assessed the clinical performance of veneered ceria-stabilized zirconia/alumina nanocomposite frameworks used for posterior three-unit fixed partial dentures (FPDs). The study revealed that this foundation material was clinically reliable with a 100% survival rate, with no damage or fractures in the veneering ceramic and positive biological outcomes of the FPDs. However, further studies with a longer observation period and larger sample sizes are necessary [100].

Borges et al. [101] evaluated the mechanical properties of composite resin filled with nylon-6 (N6) nanofibers, N6 nanofibers with MWCNTs (N6-MWCNTs) or with prepolymerized resin. These nanofillers were produced by electrospinning technique and distributed in the composite resin at 2.5%, 5%, 10%, and 20% concentrations. Addition of 10% and 20% N6-MWCNTs in composite resin showed the least polymerization shrinkage, while higher flexure strength and reduced film thickness were observed in the 2.5% and 5% groups. Saeed et al. [154] evaluated the importance of the electrospinning method in the preparation of these nanofibers. Electrospinning is a process that can produce polymer nanofibers with high porosity, gas permeability, large surface area per unit mass, and small interfibrous pore size. These properties make these nanofibers to be used in various applications number of applications such as restorative dentistry, tissue engineering, and drug delivery systems [154].

Pennisi et al. [102] compared the effect of SWCNTs and SWCNT salinized with SiO_2_ (SWCNT/SiO_2_) on the flexural resistance of indirect composite and concluded that only by incorporating SWCNTs does not improve the FS of indirect composite resin. However, addition of SWCNTs/SiO_2_ significantly improves the flexure resistance. Therefore, the silanization process plays an essential role in improving the mechanical properties of SWCNT [102]. Ali et al. [45] have discussed the most appropriate functionalization method, optimized fabrication route for synthesis, essential characterization technique, and various strategies to treat CNTs and their addition in polymer-based materials to enhance mechanical properties. Li et al. [103] evaluated the mechanical properties of CNT reinforced composites and concluded that the addition of CNT significantly increases the compressive strength and reduces the crack propagation in composite resins.

Yuksel et al. [39] conducted a study in which the epoxy matrix was supplemented with hybrid epoxy nanocomposites by incorporating combinations of nanofillers consisting of graphene nanoplatelets (GNPs) and MWCNTs in different proportions. The thermal and mechanical characteristics were examined to evaluate the impact of nanofillers present at concentrations ranging from 0.1% to 0.4% by weight. In addition, they produced and examined laminates made from epoxy nanocomposites that were reinforced with carbon fiber and modified with MWCNT/GNP. The distribution of nanofiller inside the polymer matrix was assessed using SEM. The hybrid composite, composed of a ratio of 1 part MWCNT to 3 parts GNP and a nanofiller ratio of 0.3 weight percent (wt%), exhibited superior mechanical capabilities, namely, a TS of 75.1 MPa. The inclusion of GNPs and MWCNT nanofillers in epoxy/carbon fiber laminates had a beneficial effect on the TS and strain, but had a detrimental effect on the elastic modulus. The glass transition temperature of epoxy rose from 87.25 to 114.67°C when the ratio of MWCNT to GNPs was 1:1, with a weight percentage of 0.3% [39]. Mirzamohammadi et al. [155] assessed the mechanical performance of fiber–metal laminates (FMLs) under tensile and impact conditions, finding that incorporating CNTs improved TS, elastic modulus, and impact energy, with delamination and fibrillation being key wasting mechanisms. Noticeable enhancements were reported in the nanocomposites with MWCNT:GNP (1:3 and 1:1) [155].

#### 8.1.2. Dental Adhesives

Akasaka et al. [104] examined the effects of CNT coating on the surfaces of tooth slices, viewing them macroscopically and under a scanning electron microscope (SEM), and explored how CNT coating affects the tensile bond strength of dentin adhesives. Suspending tooth slices in a CNT-dispersed solution allowed CNTs to easily adhere to their surfaces. CNTs have unique features that are suitable for biomedical purposes, according to recent studies. CNTs' ability to nucleate hydroxyapatite (HAE) has led to increased interest in their potential use in dentistry. However, only a few reports were done on the usage of CNTs as dental materials. CNTs are selectively attached to dentin and cementum surfaces, but not to the enamel surface, probably through exposed collagen (COL) fibers. Studies have found a substantial interaction between CNTs and COL fibers in an aqueous solution. The CNT–COL interface likely had a weak connection due to COL's soft nature. CNTs were unable to penetrate dentin tubules, instead forming a thin coating over COL fiber networks. Despite applying the requisite amount of CNT coating, the binding strength remained unchanged. After debonding, the adhesive remnant index (ARI) was used to classify the amount of composite left on the enamel by examining bond failure locations under a light microscope.These findings suggest that CNTs may have favorable benefits, such as hyaluronic acid (HA) nucleation and providing protection against dental bacteria, without reducing the composite strength of restorative materials. Additionally, the CNT coating did not affect the tensile bond strength of dentin adhesives, suggesting the potential use of CNTs in dental applications [104].

Marchi et al. [105] evaluated the effect of adding CNTs to two types of adhesives used in indirect bracket bonding and analyzed shear bond strength (SBS) and adhesive residue on teeth after debonding. A total of 160 bovine incisors were randomly assigned to eight groups (*N* = 20): indirect bonding with Sondhi adhesive; (2), (3), and (4), with CNT at 0.5%, 0.25%, and 0.05% concentrations, respectively; (5), with Concise adhesive; (6), (7), and (8), with CNT at 0.5%, 0.25%, and 0.05% concentrations, respectively. After etching with 37% phosphoric acid and placing the brackets, maximum SBS was evaluated using a mechanical testing machine with a 1 kN load and at a speed of 0.5 mm per minute. After bracket debonding, the ARI was used to classify the amount of composite remaining on the enamel by examining it under a light microscope to evaluate bond failure locations. NPs have been added to dental composite resins to enhance their mechanical qualities.

Turagam and Mudrakola [112] found no shrinkage of PMMA resin when 0.5 wt% of CNT was added, and a considerable reduction at concentrations of 0.25 and 0.125 wt%. No significant difference was found in SBS across Groups 1, 2, 3, and 4. There were no statistically significant differences among Groups 5, 6, 7, and 8. The SBS values in the study's eight groups ranged from 6.9 to 9.9 MPa [112].

Reynolds [156] estimated that 5.9–7.8 MPa was needed for optimal orthodontic adhesion; hence, the SBS values observed in this investigation were regarded as appropriate. The study found that adding CNT to the two indirect bonding adhesives did not improve SBS, despite being considered the best material for reinforcing resins. The use of alternative CNT (MW) or NPs of various chemical elements (copper [Cu], Ag, and gold [Au]) or even their integration into the composite resin used for bonding could be considered [105, 157].

Suo et al. [87] developed CNT coatings for the dentin surface and studied the bonding strength and the in vitro antibacterial properties of CNT-coated dentin (and *S. mutans*). Compared to MWCNTs, SWCNTs interacted with COLs more effectively. This could be because SWCNTs have more grafted reactive groups on their surface and a greater superficial area due to their reduced diameter. To create stable coatings on the surfaces, the etched dentin samples were soaked in a CNT suspension and agitated. In contrast to the MWCNT coating, the SWCNT coating's dispersion was more even. The structure of CNTs may be the cause of this phenomenon. Since MWCNTs' walls are thinner and more easily broken than those of SWCNTs, the MWCNT coating displayed few tubular structures and little dispersion after being modified with high temperatures and strong acid. To create CNTs with good dispersion, calcination, acid treatment, and carboxylation were utilized. Dispersed in deionized water, unmodified SWCNT powder, modified SWCNT powder, and modified MWCNT powder were measured for optical density (OD) using a UV–vis spectrophotometer set to 600 nm. Fourier-transformed infrared spectroscopy was used to determine the phase compositions of the unmodified SWCNTs, unmodified MWCNTs, modified SWCNTs, and modified MWCNTs. A SEM was used to characterize the morphology of the unmodified SWCNTs, unmodified MWCNTs, modified SWCNTs, and modified MWCNTs. Utilizing TEM, the particle size was examined. This study demonstrated that the SBS of dentin bonds was unaffected by CNT coating. To have specific effects on dentin bonding, modified SWCNTs and MWCNTs can, therefore, be introduced to the resin matrix like other nanomaterials to create a stable coating on the dentin surface. The homogeneous distribution and unbroken tubular SWCNTs on the dentin surface allowed SWCNTs to have greater contact with *S. mutans*. As a result, compared to the other groups, the SWCNT group had significantly less *S. mutans* cells adhered to the dentin samples. SWCNT coating on the dentin surface has the potential to limit *S. mutans* activity, however, MWCNT coating does not. As a result, SWCNT coating has the potential to be used as an antibacterial agent in the dental bonding industry [87].

Alsunbul et al. [106] added 2.5 wt% of GNPs and carbon NPs (CNPs) to a control adhesive (CA) and examined the impact of this addition on the adherence of this adhesive to root dentin and mechanical characteristics. The CNPs were procured commercially, 2.5% CNPs were included in the CA (di- and tri-methacrylate resins, PENTA [dipentaerythritol penta acrylate monophosphate], and acetone). This inclusion resulted in 2.5% CNP adhesive, while the adhesive without NPs served as a CA. The GNPs were derived from the graphite powder. Once prepared, 2.5% GNPs were added to the CA to produce a 2.5% GNP adhesive. The mechanical properties of the adhesive were strengthened by the addition of NPs. The adhesive with 2.5% GNP showed the most promising outcomes, exhibiting the highest PBS, appropriate dentin contact, and satisfactory rheological characteristics. GNP-containing adhesives may have demonstrated a stronger bond because of their larger surface area, which enables them to interact and form a firm connection with a variety of surfaces, including dentin tissue. Results are consistent with a prior study that examined a similar topic and found that adding CNPs to the adhesives increased their binding strength, although not as much as the 2.5% GNP adhesive. As demonstrated in the current study, CNPs interact and adhere selectively to dentin's COL fibers, which may have improved the adhesive's bond strength. The majority of the shortcomings found in this investigation were adhesive in nature. However, lower DC was noted for the 2.5% CNP and 2.5% GNP adhesive as compared to the CA. Previous research has demonstrated that adding NPs to adhesives can lower their DC. A higher DC of the adhesive indicates that sufficient monomers have been polymerized, which inhibits the formation of secondary caries and microleakage. Conversely, a low DC can impact a material's biomechanical qualities and shorten the lifespan of the composite restoration. This could be due to the addition of filler NPs which obstructs the curing light's pathway, which lowers the DC by producing fewer polymers. It was an in vitro study; results may vary with multiple additional challenges on the adhesive material inside the dynamic in vivo environment. In this study, only a single concentration of CNPs and GNPs was tested, highlighting the need for future research to investigate the impact of different filler concentrations on the adhesive's properties [106]. CNTs increased SBS and marginal seal in dental adhesives, with PA + CNT-modified adhesive (Group 1 B) having the lowest microleakage and maximum bond integrity. CNT-enhanced adhesives outperformed Nd:YAP laser with unmodified adhesive (Group 2 A) without sacrificing DC, indicating CNTs are effective reinforcements for durable dental restorations [158].

#### 8.1.3. Glass Ionomer Cement (GIC)

By incorporating CNTs and bioglass (BG) into GIC, Foroughi et al. [107] discovered that the cement's mechanical and bioactivity qualities were significantly enhanced. The creation of a percolating network of CNTs within the thick BG matrix led to a rise in the electrical conductivity of BG, which was also boosted after the integration of CNTs [107]. Spinola et al. [111] found that the incorporation of MWCNTs resulted in a decrease in the compressive strength of GIC while simultaneously resulting in an increase in the yield strength. On the other hand, Hamdy [159] observed that the incorporation of Ag-doped MWCNT fillers into GIC resulted in an improvement in the material's compressive strength, surface microhardness, and antibacterial action. When compared to MWCNTs, AlMufareh et al. [110] discovered that the incorporation of SWCNTs resulted in an increase in the compressive strength of the GICs. However, when compared to Ag oxide NPs, the addition of CNTs resulted in an increase in the surface roughness, but did not result in an improvement in the nanohardness. Similarly, Goyal and Sharma [109] found out that the addition of CNTs improved the mechanical capabilities of GIC; nevertheless, the color of the mixture turned black as a result of the addition. In light of this, the utilization of this mixture was suggested solely for the posterior teeth [109].

It is necessary to conduct additional research on its application in day-to-day treatment in light of the current situation, in which patients require restorations that are esthetically acceptable.

Pani et al. [108] found that the addition of CNTs to GIC cement resulted in a greater degree of color stability than the addition of AgNPs. The use of CNTs to reinforce GIC may have resulted in an enhancement in the mechanical qualities of the cement. The incorporation of CNTs with either a single or multiple walls has been demonstrated to enhance the mechanical and biological properties of GIC [108]. It is possible for the total result to be affected by factors such as the technique of preparation, diameter, concentration, and surface modification of CNTs. In order to validate the influence that CNTs have on GIC, it is necessary to have a technique that is properly planned and includes extensive characterization of CNTs as well as assessment methodologies.

### 8.2. Prosthetic Biomaterials

#### 8.2.1. Denture Bases

PMMA is a popular material in prosthetic dentistry because of its esthetic appeal, ease of manipulation, low density, affordability, and repairability [160].

Common denture base prosthesis issues include breaking owing to stiffness, produced by fatigue failure from frequent mastication or unintentional falling. To enhance the mechanical qualities of denture base, various additives such as metal wires, glass fiber, metal fillers, and CNTs have been added to the polymer. There is significant interest in utilizing CNTs in the field of prosthetic dentistry.

To compare the polymerization shrinkage of PMMA with and without microadditions of CNTs, MWCNT's were added to liquid MMA monomer in concentrations of 0.5, 0.25, and 0.125 wt% and heat polymerized using lost wax technique. The distance between the reference points in wax (before to polymerization) and acrylic (postpolymerization), as measured by a traveling microscope, was used to calculate the polymerization shrinkage of PMMA for each group. It was observed that when 0.5% of CNTs are added to PMMA resin, there is no polymerization shrinkage, while the shrinkage is lessened when 0.25% and 0.125% of CNTs are utilized in PMMA resin as compared to pure PMMA. Thus, it was concluded that microadditions of CNTs in PMMA can produce composites with the absence of polymerization shrinkage [112].

Wang et al. [65] conducted a study to examine how the mechanical properties of PMMA denture base material were affected by different concentrations of MWCNT reinforcement. It was observed that the FS and resilience values of composite resins containing PMMA and MWCNT were enhanced by the addition of 0.5 and 1 wt% MWCNT and the values were decreased by the addition of 2 wt% MWCNT. This may be caused by the nonhomogenous dispersion of MWCNTs in the PMMA matrix. The results of dynamic fatigue testing indicated that the groups containing MWCNT had low fatigue resistance. This may be due to the weak bonding between MWCNTs and PMMA at their interface where the crack propagation may intensify, resulting in poor load transfer between the two. They suggested future improvements in the form of surface modifications and silanization of MWCNTs to facilitate better interfacial bonding between CNT and polymers [65].

Mahmood [113] conducted a study that aimed to enhance the mechanical and physical properties of a high impact denture resin by using CNTs in varying amounts. Samples of high impact denture resin containing 0%, 0.5%, 1%, and 1.5% by weight of CNT were prepared and subjected to impact strength (IS), transverse strength, surface roughness, and hardness tests. According to the study, the best values for impact and transverse strength were found at 1 wt% CNT, whereas higher wt% of CNT produced lower values than those of the control group. This might be brought on by the interfacial interaction-wetting between the polymer and CNT, as well as the inhomogeneous dispersion of CNT, which leads to additional agglomerations. It is possible that the CNT particles' small size and excellent dispersion within the resin matrix may have contributed to the decrease in the surface roughness and hardness of the CNT-reinforced resin [113].

Kim et al. [64] studied the effect of incorporating carboxylated MWCNTs into PMMA (CNT–PMMA) on its drug-free antimicrobial adhesion properties against three different oral microbes (*Staphylococcus aureus*, *Streptococcus mutans*, and *Candida albicans*). MWCNT's at concentrations of 0.25, 0.5, 1.0 and 2.0 wt% were added to methyl methacrylate (MMA) monomer liquid and polymerized. The specimens were polished with either 220 or 2400 grit SiC paper to mimic scratched denture surfaces and freshly polished surfaces, respectively, and then, subjected to mechanical and microbial adhesion tests. Composites with 1% and 2% CNTs showed an increase in total fracture work; however, as the number of CNTs contained increased, other mechanical properties were gradually degraded. Lower concentrations of CNT showed enhanced microbial adhesion for both rough and polished specimens. Significantly decreased adhesion to *Candida albicans* was especially seen with 0.5% and 1% CNT specimens as compared to pure PMMA. The study concluded that CNT–PMMA was cytocompatible with oral keratinocytes and 1 wt% CNT was an optimal concentration to reduce microbial adhesion (35%–95%) of PMMA without affecting its mechanical properties and it may, thus, be utilized to develop drug free PMMA with clinically relevant antimicrobial adhesion [64].

Ghosh and Shetty [114] compared the FS of PMMA modified by fillers such as graphene oxide (GO) and MWCNTs. A 0.5 wt% MWCNT and 0.5 wt% GO were dispersed in monomer of PMMA resin by ultrasonic agitation and later heat polymerized. The results of the three-point bending test showed that Group 2 (MWCNT) had the highest mean FS of 36.5 MPa (*p*  < 0.001), followed by Group 1 (control) at 31.55 MPa and Group 3 (GO) at the lowest. This was attributed to the effective bridging of the cracks by MWCNT. Thus, it was concluded that MWCNT added at 0.5 wt% to PMMA is a simple, efficient, and cost-effective way to increase its FS and ensure durability of such prosthesis [114].

In the study by Song et al. [115], PMMA-co-polydimethylsiloxane-co-poly(hydroxyethyl methacrylate) (PMMA-co-PDMS-co-PHEMA) terpolymer (PMMA/Ter) was created and grafted to functionalize MWCNT. To create PMMA/Ter-CNT composite, the terpolymer f-CNT (Ter-CNT) hybrid was added to PMMA. Using the same process, the additional composites containing 0.5, 1 and 2 vol% of Ter-CNT were created and tested for strength toughness, thermal conductivity and self-cleansing properties. Terpolymer-grafted MWCNTs (Ter-CNT) exhibited a strong interaction with the matrix and excellent dispersion within it. When 2 vol% filler was added to the PMMA matrix, the composite demonstrated a well-balanced combination of strength and toughness, with a FS of 72.3 MPa and toughness of 10.1 MJ m^−3^, which increased by 40.1% and 578% compared with those of pure PMMA. Moreover, the composite has good self-cleaning and thermal management qualities due to its high static contact angle (110.3°) and thermal conductivity (0.50 W m K^−1^). This may be caused by the strong interfacial between the Ter-CNT and PMMA matrix, the hydrophobicity imparted by PDMS, the high thermal conductivity, and the reinforcing properties of CNT. With strong matrix contact and good dispersion, Ter-CNT filler is thought to be able to create thermally conductive routes with low CNT/matrix interfacial thermal resistance, leading to high thermal conductivity. Using molecular design, this technique created a new route for toughening and strengthening polymers that are used commonly (polystyrene, polyvinyl chloride, etc.) [115].

In a study by Mohamad et al. [116], the effects of adding 0.25 and 0.5 wt% silane coated MWCNTs and nanoglass (NG) particles alone and in combination to a 3D printed denture base resin were evaluated with respect to the printed resin's mechanical properties such as FS, IS, surface roughness (Ra), and elastic modulus. The printed samples underwent testing both prior to and following 600 cycles of thermocycling. It was observed that the 3D printed resin's FS, elastic modulus, and IS all improved as a result of the addition of NG and MWCNTs. The control group showed the least percentage drop in the modulus values, while the combination group (0.25 wt% MWCNT + 0.25 wt% NG) showed the greatest FS and least percentage change in FS and IS. The results demonstrated a greater decrease in FS for the 0.5% NG and 0.5% MWCNTs groups after thermocycling, despite the fact that FS was found to be more in these groups before thermocycling than in groups containing 0.25% fillers and the combination. The aggregation of nanofillers in the 0.5% MWCNT and 0.5% NG groups with higher filler concentrations may be the cause of this drop. Water dispersion between the polymer chains exacerbated the lower FS by releasing the bound fillers and causing areas of concentrated stress surrounding the agglomerated fillers due to the matrix's expansion and contraction during thermocycling. Thus, it was concluded that a potential alternative to traditional and milling dentures may be provided by three-dimensionally printed denture base resins augmented with a blend of 0.25% NG and 0.25% MWCNTs [116].

Alhotan et al. [88] tested PMMA–based (PMMA and MMA) denture base material with 0.05 wt% Ag-doped CNTs for IS, microhardness, and anti-candida action. The treated heat-cured PMMA–MMA with Ag-doped CNT had the maximum IS (2.2 kJ mm^−2^) compared to the control (1.6 kJ mm^−2^). Vickers microhardness (52.7 VHN) was similarly higher in the treatment group than in the control group (19.4 VHN). After 24 h, heat-cured PMMA with Ag-doped CNT had much more anti-candidal activity in the agar diffusion test than the control group. Thus, Ag-doped CNTs could be used as dental heat-cured acrylic resin fillers to strengthen dentures against sudden fractures, scratches, and candida invasion [88].

Swaroop et al. [117] assessed and compared the mechanical characteristics of heat-polymerized PMMA (polymer and monomer) reinforced with 0.5 wt% graphene, 0.5 wt% MWCNTs, and 0.25 wt% graphene + 0.25wt% MWCNTs. The samples were then subjected to Izod impact testing and 3-point bending test to test IS and FS, respectively. When nanofillers were added to the polymer, all groups showed decreased FS and IS (*p*  < 0.001). When nanofillers were added to the monomer, the groups containing MWCNTs showed enhanced FS and IS, while the groups containing graphene showed a decrease (*p*  < 0.001). Thus, it was concluded that better dispersion and cross-link formation can be achieved by mixing and adding nanofillers to the heat-cured PMMA monomer rather than polymer [117].

Aldwimi et al. [161] studied the effects of halloysite nanotubes (HNTs) and MWCNT on PMMA's TS, Vickers hardness (VH), FS, and flexural modulus. Compared to a pure PMMA matrix, the PMMA denture base composite with a blend of MWCNTs and HNTs had superior mechanical properties, including 64.4 MPa TS, 109.1 MPa FS, and 3.62 GPa flexural modulus. But VH only improved 18.93 kg mm^−2^. Using treated HNTs and MWCNTs as reinforcing materials can create innovative materials with improved properties. The mechanical qualities were considerably boosted when the ratio of HNT/MWCNT was 4.75/0.75 wt%, which may be the ideal NT filler concentration for maximal reinforcement without compromising composite mechanical properties. The mechanical strength of the composite may be reduced by agglomerating MWCNT filler particles at greater ratios up to 2.5 wt% due to their weak strength. This may cause unequal stress distribution and localized stress concentrations in the composite, which may cause cracking along the filler–matrix and filler–filler interfaces [161].

Ibrahim et al. [162] produced 3D-printed denture base resins enhanced with silanized NG and MWCNTs were tested for BFS and SBS. Before and after 600 thermal aging cycles, four groups were tested: control, 0.25 wt% NG, 0.25 wt% MWCNTs, and both fillers. Filler content and thermal cycling significantly affected BFS (*p*  < 0.001), with thermal aging having the highest impact (*p*^2^=0.551). A significant interaction between filler type, heat cycling, and denture tooth type was seen in SBS data (*p*  < 0.001, *F* = 10.340, *p*^2^=0.162). MWCNT had the highest BFS and combo had the highest SBS to 3D-printed teeth. This suggests that adding NG and MWCNTs to 3D-printed denture base resins improves their mechanical performance and bonding dependability, ensuring their long-term clinical viability [162].

Shahkar et al. [163] integrated modified MMWCNTs into PMMA denture bases, resulting in a 45% increase in FS and a 42% enhancement in modulus with a loading of only 0.25 wt%, while also boosting thermal stability (*T*_g_ = 152.7°C) and biocompatibility (153.75% cell survival). The surface modification using vinyltriethoxysilane enhanced dispersion and polymer interaction, rendering MMWCNTs a viable reinforcement for high-performance dental composites [163].

#### 8.2.2. Zirconia

Zirconia is a stable refractory oxide suitable for various applications, including crowns and bridges, implant frameworks, dental implants, and refractory equipment. Doping with 3 mol% yttria stabilizes the high-temperature tetragonal phase, improving mechanical properties like fracture toughness and crack growth resistance. However, this process may compromise material strength. Research suggests finer grain sizes can withstand aging in humid conditions. Studies on reinforcement materials' effects like CNTs on partially stabilized zirconia are also being explored [120].

Khan et al. [119] incorporated CNTs into silane primers to enhance resin cement adherence to zirconia. SWCNTs and MWCNTs were applied to 18 ZrO_2_ blanks after tribochemical silica coating. The primers were divided into five groups: Monobond S silane, 0.5 weight percent SWCNTs blended Monobond S, 0.5 weight percent MWCNTs blended Monobond S, 1.0 volume percent 3-acryloxypropyltrimethoxysilane (ACPS) + 0.5 volume percent bis-1,2-(triethoxysilyl)ethane (BTSE), and 0.5 weight percent SWCNTs blended in 1.0 volume. Multilink Speed resin composite stubs were used to bond treated ZrO_2_ surfaces. After 2 months of water storage, all primers showed increased adhesion strength. Group 5 had statistically higher EM-SBS values than Group 1 (plain silane). The study found that SWCNTs combined with ACPS and BTSE significantly improved adhesion strength [119]. Jang et al. [120] examined the mechanical properties and phase-transformation behavior of MWCNT-reinforced 3 mol% yttria-stabilized zirconia (3YSZ) composites. Using a multistage Spark Plasma Sintering technique, composites with CNT contents of 0, 1, 2, 3, 5, and 7 wt% were created. The composites showed increased fracture toughness from 4.1 to 7.0 MPa m^1/2^ with 7 wt% MWCNT/YSZ. However, the hardness decreased with increasing CNT content. The study concluded that 7 wt% MWCNT/3YSZ composite is suitable for high-impact and high-temperature applications, including environmental barrier coatings, due to its high fracture toughness. MWCNTs are a desirable alternative for SWCNTs in such composites due to their outer walls protecting inner walls [120].

Son et al. [121] investigated the impact of YSZ and CNTs on the mechanical characteristics and oral rinsing stabilities of commercially available photocurable DRCs. The composites were produced using digital light processing and analyzed for their rheological behavior. The results showed that adding CNT increased the strength to a maximum of 41.91 MPa for 0.3 wt% addition, but it dropped as the CNT concentration increased. The CNT-added samples had significantly lower hardness values than the YSZ-added specimens. The study concluded that DRCs containing 0.5 wt% YSZ had increased hardness, FS, and oral rinse stability compared to those containing CNT, suggesting YSZ may be a more suitable ceramic additive for improved DRCs [121]. da Silva et al. [122] compared the microstructure, FS, fracture toughness, and optical properties of Y-TZP/MWCNT-SiO_2_ to standard Y-TZP. The precursor hydroxide coprecipitation, hydrothermal coating of CNTs, and dispersion in commercial Y-TZP powder created the Y-TZP/MWCNT-SiO_2_ nanocomposite. Silica functionalized Y-TZP/MWCNT-SiO_2_ had an opaque-white color and good optical properties for dental restorations. The Y-TZP/MWCNT-SiO_2_ did not differ significantly from the control Y-TZP in VH (10.14 ± 1.27 GPa; *p*=0.25) and fracture toughness (4.98 ± 0.30 MPa m^1/2^; *p*=0.39). The FS of Y-TZP/MWCNT-SiO_2_ nanocomposite was lower than the control (623.7 ± 108.8 MPa; 299.4 ± 30.5 MPa; *p*=0.003). The Y-TZP/MWCNT-SiO_2_ composite developed in this study had good optical properties, but the coprecipitation and hydrothermal treatment methods should be improved to prevent strong agglomerates and porosities from the bundles and particles, which may reduce the material's FS [122].

### 8.3. Bone Regeneration

Advances in regenerative medicine enable tissue regeneration using cells, growth factors, and scaffolds. Among these three aspects of regenerative medicine [164], scaffolds have garnered interest and led to the creation of many scaffolds for bone and cartilage regeneration [165]. Bioabsorbable polymers, composites, and ceramics have been studied as scaffold materials for bone regeneration medicine and CNTs are being studied more [166].

Zanello et al. [123] investigated SWCNT and MWCNT with and without chemical changes to determine which kind best supports bone growth. SWCNTs with neutral electric charge produced plate-shaped crystals comparable to HA crystals in woven bone and had the highest cell proliferation. However, negatively charged and zwitterionic SWCNT cell counts were much lower, demonstrating that electric charges do not promote osteoblast proliferation. Cells cultured on neutral CNTs with sustained osteoblast membrane electrical activity and increased Ca^2+^ channel functionalities showed biocompatibility as prepared AP, AP-SWNTs and AP-MWNTs. In osteoblasts cultivated on MWNTs, plasma membrane functions and cell shape changed dramatically. More research is needed to understand how nonbiodegradable CNTs affect the immune system. AP- and f-CNTs' hydrophilicity, hydrophobicity, topology, and impurities need further investigation. Application of CNTs could serve as suitable scaffold materials for osteoblast proliferation and bone formation [123].

Gholami and Noor [124] conducted a study to examine the mechanical strength and cytotoxicity of HAE/MWCNTs/bovine serum albumin (BSA) composites with different types of MWCNTs including hydroxylated and carboxylated MWCNTs (MWCNTs-OH, MWCNTs-COOH). It was observed that HA/MWCNTs-COOH/BSA showed the highest compressive strength. This is due to the stronger bond that exists between MWCNTs-OH and MWCNTs-COOH with HA particles in composites that use f-MWCNTs. f-MWCNTs provide a homogenous dispersion of MWCNTs in the HA matrix, hence, improving the distribution of stress in the composite structure, resulting in uniform strengthening. All developed composites at low concentrations did not cause cytotoxic effects on cell proliferation and HA/MWCNTs-COOH/BSA composites showed the highest viability. This shows the cell proliferative effect of the HA/MWCNTs-COOH composite as evidenced by a higher metabolic activity of the fibroblasts. However, when the concentration was increased, it showed a reduction in cell viability. This is due to the high tendency of non-f-MWCNTs to form clumps and cause aggregations, whereas the f-MWCNT composites have better dispersion. The clumps cannot be completely phagocytized by macrophages and will increase the cytotoxicity effect. Cell proliferation of the compound hints at potential wound healing effects which add further benefits to this composite. More studies are needed to investigate on the mechanism of affecting cells by these composites, and the compressive strength of composite to make it applicable in clinical use, under load-bearing [124].

Tanaka et al. [125] investigated the use and efficacy of scaffolds made of MWCNT blocks and rhBMP-2 in bone regeneration. Ectopic bone development on MWCNT blocks containing rhBMP-2 had a maximal compression strength equivalent to cortical bone. Stress resistance is greater in MWCNT blocks with rhBMP-2. Despite bone development, PET-reinforced COL sheets failed nonbrittlely at lower stress than MWCNT blocks. This shows that bone fractures differently around MWCNT blocks and PET-reinforced COL sheets. MWCNT loaded with rhBMP-2 had equivalent bone marrow density to PET-reinforced COL sheets. The rhBMP-2-loaded blocks can fill bone deficiencies and scaffold bone regrowth [125].

de Moura et al. [126] conducted a study to investigate the biological, physical, and antimicrobial characteristics of poly lactic acid (PLA) porous membranes. They also examined the combined effect of reinforcing these membranes with various concentrations of BG and CNT in guided bone regeneration (GBR) using a controlled humidity method. The pore geometry of PLA/BG/CNT undergoes a transformation from its initial sphere-like form to irregular morphologies. Incorporating 5 wt% BG results in an augmentation of the surface porosity and biological characteristics of the PLA, rendering its surface conducive to the deposition of HA. CNT exhibited antibacterial activities without affecting the bioactive characteristics of the compositions. PLA/BG/CNT promotes cell growth and division without causing any harm to the cells. The combined effect of BG and CNT on PLA porous membranes is particularly noticeable in the case of PLA/5BG/1.0CNT, which exhibits significant bioactivity, antibacterial properties, and exceptional biocompatibility. The PLA/BG/CNT membrane has promising prospects for directed bone regeneration; however, further in vivo experiments are necessary to validate this idea [126].

Sanchez et al. [127] studied the characteristics and compatibility of chitosan-HA-MWCNTs (CS-HA-MWCNT) films at various concentrations. A biocompatible, mechanically sound, and flexible patch was desired. Particle-CS matrix interactions caused filler clumping, affecting material quality. Nanocomposite films' mechanical characteristics are enhanced due to the homogenous distribution of 5 wt% HA NPs and 0.5 wt% MWCNT. CS-HA-MWCNT film with 30% HA NPs and 3% MWCNT was nonuniform. HA-MWCNT interaction clusters fillers above 1 wt%. CS-HA and CNT films of 5% and 0.5% by weight conduct like bone and are biocompatible with osteoblasts. HA NPs aggregate and diminish cell viability, although higher concentrations (30% and 50%) increase cytotoxicity. MWCNT at 3% weight percent impairs cell survival due to toxicity [127].

Silva et al. [128] investigated the impact of CS/CNT scaffolds and the low-level laser technique (LLLT) on bone defect regeneration. The porosity of CS/CNT scaffolds remains consistent following mineralization. Mineralized CS/CNTs with LLLT (C + CNTsM + L) scaffold bone formation improved density, thickness, and volume. CS/CNT scaffolds were biocompatible, with no inflammation or fibrosis. C + CNTsM + L also had more ordered osteocytes and stronger osteocalcin (OC), osteopontin (OP), and vascular endothelial growth factor expression. CS-based CNT materials with LLLT improved bone healing [128].

Yikun et al. [167] created a robust and hydrophilic CNT–based membrane for GBR by dispersing SWCNTs in HA. The membrane preferentially stimulated osteoblast proliferation while protecting nonosteogenic cells, hence, facilitating bone development in rat calvarial defects without CNT diffusion. Its superior strength and biocompatibility render it a promising candidate for clinical GBR applications, contingent upon additional safety evaluations [167].

Diao et al. [168] constructed a 3D-printed scaffold composed of PLA-nano-HAE with integrating osteogenic CNTs with antibacterial chlorhexidine (CHX; PLA/nHA/CNTs-CG). The scaffold exhibited regulated porosity, sufficient mechanical strength (8.44 MPa), superior biocompatibility, improved cell adhesion, and osteogenic differentiation, while successfully suppressing *S. aureus* and *E. coli*. This dual-functional architecture facilitates both bone regeneration and infection management in defect repair [168].

### 8.4. Tissue Engineering

CNTs have emerged as promising nanomaterials in tissue engineering owing to their remarkable mechanical strength, electrical conductivity, and biocompatibility. Their distinctive architecture promotes cell adhesion, proliferation, and differentiation, rendering them significant in tissue engineering. CNTs enhance scaffold efficacy by bolstering mechanical stability and facilitating stem cell proliferation and tissue regeneration. Nevertheless, issues including toxicity and long-term biocompatibility necessitate additional studies to enhance their therapeutic applicability in regenerative medicine [169].

Chen et al. [129] conducted a study to examine in situ precipitation as a new method to prepare CS–MWCNTs/HAE nanocomposites with good biocompatibility and high strength. It was observed that HA particles and MWCNTs in the polymer matrix were uniformly distributed. An increase of multiwalled carbon/CS weight ratios from 0% to 5% led to a sharp increase in the elastic modulus and compressive strength, indicating the enhanced mechanical properties of the composites. The results of cell morphology and proliferation indicated good attachment and adhesion of preosteoblast cells on the surface of the CSMWNTs/HA composites and possess noncytotoxicity, which proved good biocompatibility. In conclusion, CS-MWNTs/HA nanocomposites exhibited great mechanical properties, biocompatibility, and bioactivity which is a potential biomaterial for bone tissue engineering [129].

Ignat et al. [130] conducted research to examine how the versatility of human adipose-derived stem cells (hASCs) was affected during adipogenesis and osteogenesis by different concentrations of GO and CNTs reinforcement in a cellulose acetate (CA) membrane. It was observed that porous morphologies with open and interconnected voids in the micro-CT analysis of all samples. It was observed that the total porosity and structural thickness of the CA membrane were minimum by the addition of 1.0 wt% GO–CNT enrichment, with larger and more homogenous pores and thicker walls which improved mechanical stability. However, 0.5 wt% increased the total porosity and structural thickness of the membrane. This may be due to 0.5 wt% exceeding the threshold value, whereby the repulsion kinetics between the hydrophobic nanomaterial and nonsolvent generated enough energy to engage with large volumes of the polymer. The results of cell adhesion via immunofluorescence staining indicated that an increase in GO–CNT concentration improved hASCs adhesion and cytoskeleton formation. The adipogenic and osteogenic differentiation properties of hASCs increase proportionally with the GO–CNT concentration. It is suggested that CA–CNT–GO films can be utilized in tissue and stem cell engineering based on the great potential of versatility and selective cell differentiation [130].

Fathy et al. [131] conducted a study to examine how the mechanical properties and biocompatibility of carbonated HA derived from cuttlefish bone were affected by the addition of f-CNTs and CS composite. The uniform distribution of f-CNTs on the HA maintains the HA surface roughness without affecting the bioactivity and osteoconductivity. The result demonstrated that the elastic modulus and compressive strength were enhanced by HA/CS/f-CNTs at low strain, but the value was reduced in high stress due to brittle failure and collapse of the microstructure under compression. HA group showed the highest osteogenic ability and MSCs proliferation [131].

Farshidfar et al. [132] studied the effects of MWCNTs and curcumin (CUR) reinforcement on the physical, chemical, and biological properties of a 3D scaffold containing COL. They found that MWCNTs up to 1% increased TS and elasticity, but decreased with more than 1 wt%. Adding up to 10 wt% CUR further enhanced TS and HA crystal formation. The COL–MWCNTs 1%-CUR 10% composite scaffolds showed excellent surface wettability and decreased porosity. The addition of 10% CUR reduced tissue inflammation and increased vascularization. The authors recommend further tests to evaluate CUR's potential as an osteogenic agent in bone thinning [132].

Wu et al. [170] illustrated that MWCNTs markedly improved bone tissue engineering scaffolds. At a 1% loading, MWCNTs enhanced mechanical strength, elasticity, and osteoinductive activity while preserving biocompatibility. In vitro assessments with human bone marrow mesenchymal stem cells and in vivo trials on rabbit femurs validated improved osteogenesis and screw stability, demonstrating the promise of MWCNTs in the development of high-performance bone regeneration scaffolds. Their combination mitigates significant constraints of single-polymer systems, presenting a potential approach for functionalized bone tissue synthesis [170].

### 8.5. Periodontal Treatment (Gum Care)

Oral bacteria in biofilms cause periodontitis, an infectious-inflammatory condition that often leads to tooth loss. Plaque and calculus removal is beneficial in many instances; however, tissue-invasive organisms can repopulate and induce recurrence in advanced periodontal disease and aggressive periodontitis [171]. Nanotechnology has facilitated drug delivery in periodontal treatment. The emergence of nanodelivery systems has significantly advanced the field of periodontics [172]. A variety of treatments exist for the management of periodontal diseases, encompassing both pharmacological therapies and surgical procedures. Medicinal therapies consist of medication molecules that are naturally large particles, making it difficult for them to infiltrate the periodontal pockets. In contrast, the nanoscale dimensions of NPs facilitate their access to subgingival areas [173, 174].

For recurrence and spread, adjunctive treatment have been recommended [175]. CNTs have been experimented for the treatment of periodontal diseases. Mei et al. [133] investigated how adding MWNTs/HAE NPs (MWNTs/HA NP) to poly(L-lactic acid) (PLLA) electrospun membrane to create a guided tissue regeneration (GTR) membrane affects its biological properties. PLLA/MWNTs/HA generated a three-dimensional porous framework with varied fiber diameters and a more beaded structure, encouraging GTR angiogenesis. HA NP also slowed PLLA membrane breakdown and pH lowering. The release of OH^−^ neutralizes PLLA degradation acid. PLLA/MWNTs/HA membrane was more selective than controls, increasing periodontal ligament cell (PDLC) adherence and proliferation by up to a third and decreasing gingival epithelial cell (GEC) adhesion and proliferation by a third. Higher energy and a varied material surface may promote osteoblast adhesion and decrease competing cell adhesion. A single-layered material, PLLA/MWNTs/HA, stimulated human PDLC (hPDLC) growth and adhesion, but inhibited GECs [133].

Suo et al.[134] examined how the osteogenic properties of composite scaffolds were affected by different concentrations of GO/HA/CS reinforcement using 3D printing technique. The authors created a multilayered interconnected GO/HA/CS scaffold with a pore size of 450–580 μm. Increasing concentrations of GO reduced the porosity and enhanced the structural mechanics of the scaffold such as water swelling, degradation rate, and elasticity. Appropriate amounts of GO such as 0.25% was found to be noncytotoxicand promoted cell growth and proliferation with excellent biocompatibility with osteoblasts which helps in the repair of bone defects [134].

Zarei et al. [135] examined how the physical, chemical, and biological properties of poly(3-hydroxybutyrate) (PHB) electrospun scaffold are affected by the incorporation of 1% CNT for periodontal regeneration. Results showed the mechanical properties and TS of PHB/1%CNTs were enhanced, similar to hPDL. PHB/1%CNTs formed a scaffold akin to COL fibrous structure in the PDL, with increased fiber diameter and porosity. f-CNTs improved the wettability, bioactivity, and in vitro and in vivo biocompatibility. In vitro, the biocompatibility study showed a significant increase in the attachment and proliferation of the PDLSCs for PHB/1%CNT scaffolds than PHB controls. On the other hand, results from in vivo demonstrated that CNTs in the scaffolds triggered mild foreign body type giant cell reaction and inflammation as well as moderate vascularization [135].

Study by Suo et al. [176] on 3D-printed CNT/CS/sodium alginate (CNT/CS/AL) composite scaffold exhibited superior mechanical strength (18–80 kPa elastic modulus), enhanced biocompatibility for hPDLSCs, and significant antibacterial efficacy against *P. gingivalis* (30% inactivation at ≥0.5% CNT). This scaffold showed promise for periodontal tissue regeneration since it has structural stability, supports cell growth, and has antibacterial qualities that make it useful for advanced periodontal therapy [176].

The MWCNTs have shown significant antibacterial efficacy against principal periodontitis pathogens (*Prevotella intermedia* and *Aggregatibacter actinomycetemcomitans*), causing cell wall disruption, lysis, and ghost cell generation at low concentrations (5–10 µL) with extended exposure (72 h). TEM validated the internalization and structural damage of bacterial cells, underscoring the promise of MWCNTs as a nontoxic and compatible NT for addressing gum disease [177].

### 8.6. Endodontic Treatment

Complete bacterial eradication and gutta percha sealing determine root canal therapy success [178]. As root canal cleaning and shaping may not remove bacteria, chemical biomaterials improve results. Sodium hypochlorite, CHX, and quaternary ammonium silane can supplement mechanical cleaning [179].

Sanaee et al. [136] conducted research to examine the effect of particle size distribution and physical properties of mineral trioxide aggregate (MTA) with reinforcement of different concentrations of MWCNT. It is shown that the compressive and FSs were increased in 0.25, 0.50, and 0.75 wt% MWCNTs reinforced MTA specimens, but reduced in 1.0wt% MWCNTs–MTA. This is because of the non-uniform dispersion of MWCNTs due to a higher amount of MWCNTS being utilized. The addition of MWCNTs improved the mechanical properties of MTA significantly without impairing the setting time. MTA setting time is accelerated by decreasing particle size distribution without any addition of chemicals. Hence, MWCNTs modified MTA material with superior mechanical strength and short setting time is developed. More investigations are needed to study the mechanism of the MWCNTs effect on MTA material [136].

Baghdadi et al. [137] conducted research to examine the effect on physiochemical properties of BioRoot root canal sealer due to reinforcement of different concentrations of nanomaterials including MWCNTS, titanium carbide (TC), or boron nitride (BN) in different weight percentages (1 and 2 wt%). The physiochemical properties of 1 wt% composites were acceptable due to the smaller particle size of the nanomaterial which was more homogenously distributed in the Bioroot matrix. The initial and final setting times of 1 wt% composites were significantly shorter than BioRoot RCS and 2-wt% composites. Bioroot/1 wt%BN had the shortest initial setting time, but Bioroot/1 wt% CNT had the shortest final setting time. However, in the 2 wt% composites, the initial setting times were longer, but the final setting times were shorter when compared with BioRoot RCS. The solubility and elution profiles of the composites are lower with increasing pH over time. The pH value of 1 wt% composites is higher than the 2 wt% composites with Bioroot/1 wt% TC showing the highest alkaline pH value. Hence, a bioceramic-containing RCS with more alkaline, quicker setting, and lower solubility could be developed [137].

Another similar study Baghdadi et al. [138] conducted a study to examine the microstructural properties and compressive strength of BioRoot RCS with reinforcement of various concentrations of MWCNTs and TC nanopowder. The 1 wt% Bioroot/MWCNTs and Bioroot/TC improved significantly in the compressive strength whereas the 2-wt% composites did not show any increase. A 1 wt% Bioroot/MWCNT and 1-wt% Bioroot/TC showed uniform arrangement of particles and well interaction between hydration products and NPs, thus, increasing the density and bonding strength. As the concentration of MWCNT and TC increases, more cracks and fractures are evident on the surface indicating brittleness. BN reinforced BioRoot RCS did not enhance the compressive strength or its microstructure. The addition of 1 wt% MWCNT and TC can enhance the mechanical properties of BioRoot. Further studies are needed to study the chemical composition, physiochemical, mechanical properties, and biocompatibility of the new composites [138].

Marica et al. [139] studied the physicochemical, structural, and morphological properties of a nanomodified endodontic sealer. The sealer was modified with MWCNTs, CHX, and colloidal AgNPs. The nanomaterials were distributed evenly, and the sealer showed improved nanomechanical properties. The modified sealer showed better antimicrobial activity against E.faecalis and *S. aureus*, and was well-adapted to root canal dentin, indicating its potential in clinical endodontics [139].

Wang et al. [180] illustrated that the CNTs significantly facilitated mineralization in human cementoblasts (HCEM) without inducing cytotoxicity, hence, augmenting calcium deposition and upregulating osteogenic genes (alkaline phosphatase [ALP] and BSP). CNTs affixed to cellular surfaces indicate their potential as a bioactive element for apical periodontal regeneration materials [180].

### 8.7. Dental Implants

Dental implants are artificial prosthetics used to replace damaged or infected teeth, improving a patient's quality of life. Common materials include titanium and zirconia. However, each has limitations due to osseointegration and mechanical properties. The high cost of dental implant technology limits its accessibility. CNTs are suitable for dental implant coverings because of their biocompatibility, osteoconductivity, and mechanical strength. Their unique features improve implant longevity, osteoblast adhesion, and bone deposition. CNTs can also be mixed with nano-Ag to make dental antibacterial coatings. Better mechanical, chemical, and biological properties have boosted their use in dental implant performance and reliability [181, 182].

Recent research focuses on developing new materials that adapt to the oral environment, with CNTs being considered a unique material with remarkable properties [183, 184].

Dental implants endure significant stress and forces from mastication or oral conditions, such as bruxism. Effective management of biomechanical stress on dental implants necessitates the consideration of compression, tension, and shear forces, together with the functional surface area responsible for load dissipation. CNTs are a distinctive material characterized by exceptional mechanical strength, minimal water permeability, and superior adsorption capabilities. They demonstrate an average Young's modulus (1 TPa) and are analogous to the TS of diamonds. CNTs are frequently utilized in novel dental implant materials to improve compression, shear, and tensile strain resistance. The efficient reinforcing of bulk material via CNT is contingent upon aspect ratio, dispersion, symmetry, and stress transmission at the CNT–matrix connection. Well-dispersed and randomly oriented CNTs are favored for their anisotropic mechanical characteristics [185, 186].

**Figure 4:**
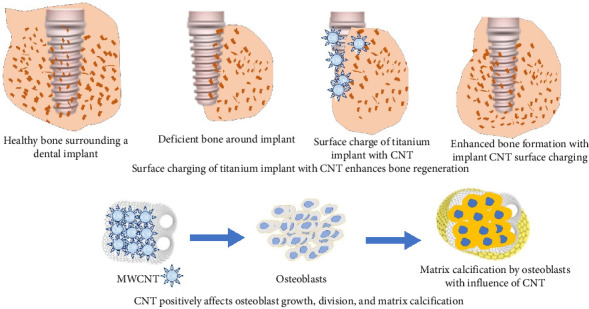
Impact of CNT surface coating on titanium surfaces enhances bone calcification.

CNTs have been utilized for surface charge adjustment in titanium implants and evaluated for strength as a measure of osseointegration. A study examining the impact of bone tissue formation on CNT–Ti composites [43] found that cell growth on the CNT–Ti composite was comparable to that of pure titanium. The initial cell dispersal occurred more rapidly on the pure titanium layer than on the CNT–Ti hybrid, attributable to its roughness. Cell development was superior on the CNT–Ti composite, as seen by increased deposits of calcium on the CNT–Ti composite compared to pure titanium. The elevated activity of ALP detected on the CNT–Ti composite indicates that the cells began to differentiate sooner on this surface. Thus, CNT–Ti composite was nontoxic, as no inflammatory reaction was found during the experiment [187]. This impact of CNT on titanium surface is depicted in Figure 4. Subramani et al. [188] demonstrated that enhancing the hydrophilicity of CNTs (by including COOH groups) on CNT-layered titanium surfaces positively affects osteoblast growth, division, and matrix calcification [188]. The hydrophilic characteristics contributed to enhanced cytocompatibility compared to MWCNTs [189].

CNT coating on zirconia implants enhances cell attachment due to surface roughening effects. However, increased CNT content does not improve implant toughness due to the absence of a well-dispersed CNT network, which inhibits its toughening effect. A homogeneous distribution of CNTs is crucial for enhancing implant toughness [190].

The in vivo results of the study conducted by Khan et al. [191] indicated that CNT-coated dental implants markedly improved osseointegration and bone regeneration. In a rabbit femur model, the CNT-modified implants demonstrated superior bone-implant contact, increased new bone formation, and higher biomechanical stability relative to uncoated titanium implants. Histological research verified the presence of more mature and denser bone tissue around CNT-coated surfaces, signifying enhanced biological performance and integration. The results indicated that CNT coatings could serve as an effective surface modification approach to enhance the clinical success of dental implants [191]. The research by Patel et al. [192] indicated that CNT-coated biopolymer nanofibers markedly improved tissue repair and bone regeneration in vivo. In a rat subcutaneous model, the CNT-coated scaffolds decreased inflammation by downregulating pro-inflammatory cytokines like IL-6, TNF-α, and macrophage recruitment, while enhancing angiogenesis through enhanced neovascularization and vWF expression. In a calvarial bone defect model, the CNT-coated nanofibers enhanced bone regeneration, exhibiting increased bone volume, mineral density, and expression of osteogenic markers (BMP-2, OCN, and OPN) relative to controls. These findings underscore the scaffold's capacity to facilitate anti-inflammatory, pro-angiogenic, and osteogenic responses, rendering it a suitable platform for bone regeneration [192].

Elleuch et al. [193] elucidated the enhancement of adaptability in strengthened nanocomposite composites for dental implants through the incorporation of CNTs into a titanium matrix. A detailed mathematical model of the micromechanics simulation was developed and refined, and a three-dimensional model of the bone structure surrounding the implant was created. Finite element analysis (FEA) was used to evaluate the impact of CNT–Ti implant on osseointegration and bone remodeling of the bone over a period of 48 months. The study assessed the impact of the CNT–Ti implantation on temporal performance, analyzed stress distributions and micromotions in jaw bones, and examined three agglomeration types. The skeletal system's mechanical response to the CNT-reinforced dental implantation was anticipated. The research determined that the CNT aggregation of CNTs diminishes the elastic stiffness of the matrix [193]. Esmaili et al. [145] aimed to enhance the corrosion resistance of the Ti-6Al-4V implant and reduce the harmful ion release by coating the alloy with a tantalum carbide (Ta_2_C)/MWCNTs multilayer thin film. The Ti-Ta_2_C/MWCNT surface showed increased hardness, improved corrosion resistance, and reduced ion release. The load–displacement curve shifted to the left and the final depth decreased due to a 4.8-fold increase in the nanohardness of the Ti-Ta_2_C/MWCNT sample compared to the Ti-6Al-4V sample. The Ta_2_C/MWCNT coating layer improved the fracture toughness, tribological properties, and the resistance to plastic deformation, with higher hardness attributed to the uniform distribution of MWCNTs on the Ti substrate, which prevented dislocation movement in the coating. The increased corrosion resistance of Ti-Ta_2_C/MWCNT sample is attributed to the lower porosity of Ta_2_C/MWCNT compared to other samples [145].

Malekahmadi et al. [146] aimed to optimize the combination and temperature of synthesized HAE CNT–water hybrid nanofluid, aiming to achieve both biocompatibility and thermal conductivity for dental implant coatings. HAE was synthesized, and then, the prepared CNTs were added to the mono-nanofluid of HAE (0.2, 0.4, 0.6, 0.8, and 1.0 vol%), resulting in a hybrid nanofluid with a 1:1 volume ratio of 1:1 for of HAE/CNT. The results showed a 25.83% increase in thermal conductivity for the 1.0 vol% composite sample of CNTs at 50°C compared to mono nanofluids. The findings suggest the increased effect of CNTs on enhancing thermal conductivity rather than HA's influence in decreasing it [146]. Vijay et al. [194] reviewed about carbon nanomaterials modified biomimetic dental implants for diabetic patients. The use of biomimetic dental implants may enhance the success rate of implants for diabetic patients, and their antibacterial properties may help in preventing peri-implantitis. Additionally, carbon nanostructures in drug delivery systems may facilitate drug transport and mimic protein channels, which could improve wound healing and thereby reduce dental implant failures [194]. Sivaraj et al. [147] investigated the antibacterial properties of Cu-HAE/f-MWCNT composite coated heterogeneous implant surfaces against gram positive and gram-negative microorganisms and the potential effect on surface corrosion effects occurring in stimulated body fluids. The corrosion current density dropped dramatically from 6.8 to 3.8 μA, indicating that the Cu-substituted HAE/f-MWCNT composite coating had better barrier qualities. When compared to HAE/f-MWCNT, the hybrid Cu-HAE-MWCNT composite demonstrated superior antibacterial activity against gram-positive and gram-negative bacteria, with a maximal inhibition zone of 13–17 mm. When compared to other microorganisms, the Cu-HAE/f-MWCNT nanocomposites demonstrated good antibacterial activity against *E. coli*. The abnormal form of the bacterial cell's morphology could be caused by the permeability barrier malfunction of the nanosized Cu substituted HA/f-MWCNT composite, ultimately leading to the cell's demise. The Cu-HAE/f-MWCNT nanocomposite demonstrated the nontoxic and biocompatible nature of the coated material, making it appropriate for use in biomedical applications [147].

In vivo study by Facca et al. [195] demonstrated that CNT-reinforced HAE coatings on titanium implants promoted normal bone development and enhanced osseointegration rates relative to conventional HAE alone. It observed that incorporation of CNTs does not provoke cytotoxicity, confirming their safety for in vivo applications. The mechanical integrity of the bone-implant interface was preserved, which is essential for the enduring success of dental implants [195].

Logesh et al. [196] performed addition of hydroxylated MWCNTs into HAE coatings using plasma electrolytic oxidation (PEO) improved the surface characteristics of titanium–niobium–zirconium (TiNbZr) dental implants. MWCNTs enhanced surface roughness, wettability, and corrosion resistance, while concurrently diminishing elastic modulus for increased mechanical compatibility. The altered coating exhibited significant antibacterial properties and enhanced osteoblast cell viability, rendering it a promising candidate for long-lasting and bioactive dental implants [196].

Logesh and Choe. [197] integrated MWCNTs into the PEO-derived Ag/HA coating for Ti-6Al-4V dental implants improved mechanical qualities, corrosion resistance (*R*_*p*_ = 3.25 × 10^15^ Ω cm^2^), and antibacterial effectiveness. In conjunction with AgNPs, MWCNTs enhanced a multifunctional surface that augments implant durability and performance, showcasing their promise in sophisticated dental implant coatings [197].

### 8.8. CNT Reinforced Composite Materials

CNT composites are being explored for their potential applications in dentistry, enhancing the mechanical properties and biocompatibility of different biomaterials demonstrating their adaptability and efficacy in tackling prevalent issues in dentistry practices. Teh and Lai [183] engineered CNT based nanocomposites to enhance osseointegration and stability of dental implants, mitigating concerns associated with mechanical incompatibility with natural bone.

The surface functionalization of CNTs demonstrated potential in improving the biocompatibility and mechanical characteristics of these composites, which is essential for effective implant integration [183]. Abazari et al. [198] observed the structural and biological potential of CNT reinforced magnesium (Mg) matrix composites. Mg-9Al-0.4 wt% CNT composites have 18% better ultimate TS and 150% higher elongation due to load transfer and grain refinement. Advanced fabrication technologies include powder metallurgy, spark plasma sintering, and surface functionalization address CNT aggregation and weak interfacial bonding. As solid lubricants, CNTs improve wear resistance but promote corrosion in Mg composites by galvanic coupling. Biocompatibility and scalability concerns hinder clinical applicability despite encouraging in vitro results. To improve these composites for industrial and biomedical usage, CNT alignment, hybrid reinforcements, and corrosion-resistant coatings needs to be investigated [198].

To improve body corrosion resistance, Bakhsheshi-Rad et al. [199] created a smart dual-layer Mg implant covering with ZnO and CNTs (MWCNTs). The inner ZnO layer (1.1 μm thick, NPs) served as a base, while the outer MWCNT layer (10.2 μm thick NTs) provided additional protection. This dual coating reduced corrosion current by 96% compared to bare Mg and maintained strength after soaking in body-like fluids. Concurrently, single-layer ZnO coatings underperformed, whereas uncoated Mg exhibited significant corrosion [199].

Tian et al. [200] grafted CS onto MWCNTs to create composites with improved biological properties. FTIR spectroscopy confirmed covalent bonding, and thermal analysis showed 38% CS. MWCNT–CS composites increased microtensile bond strength and cell survival, making them suitable for dental applications, mineralization, bone scaffolds, and medication delivery [200]. Son et al. [121] included CNT into DRCs to enhance mechanical qualities, including hardness and FS, essential for durability in restorative applications. They also showed that resin based composited containing CNT improved stability during oral washing, rendering them appropriate for prolonged application in the oral cavity [121]. Goyal and Sharma [109] explored the use of reinforcing GIC with MWCNTs to enhance its mechanical performance for posterior restorations. The composites were examined using various techniques, revealing that MWCNTs positively influence GIC's performance, making it a more durable and effective option for tooth restoration [109].

Al-Allaq et al. [201] investigated MWCNT-reinforced HAE/high-density polyethylene (HAE/HDPE) biocomposite produced using hot-pressing. FE-SEM and atomic force microscopy demonstrated a homogeneous distribution of MWCNTs and enhanced microstructure, whereas thermal analysis indicated a 35% increase in crystallinity compared to pure HA/HDPE. The improved characteristics render these composites advantageous for bone reconstruction applications.

Al-Harbi et al. [202] investigated addition of SWCNTs to a hybrid bioactive nanocomposite membrane (CA-HAP-BG-SWCNTs) for dental use. Casting dissolution was used to make the nanocomposites, which were characterized by XRD, FTIR, SEM-EDX, and Raman spectroscopy to demonstrate their compatibility. The membrane's semicrystalline structure and mechanical and thermal properties were improved by SWCNTs. The nanocomposites had modest to noncytotoxic effects on Vero cells, even at high concentrations, making them attractive for dental applications. They had no antibacterial activity against tested pathogens. SWCNTs in the membrane may improve dental material biocompatibility and mechanical reinforcement [202].

Zhao et al. [203] created a 3D-printed bone scaffold utilizing PCL fortified with carboxylated MWCNTs and bacterial cellulose (BC). The 1 wt% MWCNTand BC composite scaffold had the best shape, with a high compressive strength of about 86 MPa and great osteogenic function. In vitro and in vivo experiments showed that MWCNTs are biocompatible, that they help cells adhesion, and that they can effectively repair mandibular defects. This demonstrated that MWCNTs could be useful for therapeutic applications that involve bone regeneration [203].

Alani et al. [204] investigated MWCNT-reinforced HAE, HDPE (HAE/HDPE) biocomposite for osseous repair. Hot-pressed scaffolds improved bone tissue regeneration in rabbit femur defects, exhibiting increased bone density and reduced adipocyte presence compared to controls. Results affirm that MWCNTs enhanced structural and mechanical qualities, facilitating bone regeneration [204].

## 9. Critical Analysis on Use of CNT for Dental Applications

### 9.1. Structural Analysis on Use of CNT for Restorative Biomaterials

#### 9.1.1. Composite Resins

Multiple research projects have assessed the mechanical characteristics, production, and characterization of composite resins reinforced by CNT. The mechanical properties of CNT reinforced composite resins were determined by key aspects such as the kind of nanomaterial, production technique, functionalization, surface treatment, homogeneity, and dispersion of CNTs inside the resin matrix [99, 100, 103, 154]. Furthermore, it is crucial to address the monitoring of properties of polymer composites including CNTs, controlling the structure of the material, characterizing the particles of NTs, understanding their aggregation, and exploring the utilization of single, double, or MWCNTs. Hybrid nanofillers is reported to synergistically improve the mechanical properties of hybrid nanocomposites. The amount of fillers significantly influences the distribution and agglomeration processes within the polymer matrix. The microscopy results verified the effective distribution of fillers in MG13 within an epoxy matrix, which was found to result in the highest mechanical performance. However, it was shown that certain GNPs tended to form aggregates when combined with different filler ratios. The interplay between the epoxy matrix and the combination of fillers led to an elevation in the glass transition temperature (*T*_g_) and a reduction in the onset temperature (*T*_o_), potentially impacting the application of these composites. Hence, it is important to conduct more inquiries, taking into account various proportions of MWCNT:GNP and including a slightly higher amount of filler [39].

#### 9.1.2. Dental Adhesives

Akasaka et al. [104] examined the surface changes induced in the tooth slices caused by applying a CNT coating. It was observed that CNTs selectively adhered to the dentin and cementum surfaces, possibly due to their attachment to the exposed COL fibers. These findings align with those of previous studies, which have reported a strong interaction between CNTs and COL fibers in an aqueous environment [205–207]. In addition, the CNT coating did not affect the tensile bond strength of dentin adhesives, suggesting that CNT coatings could be a possible application in dental materials. Marchi et al. [105] assessed the impact of CNT addition to to two types of adhesives used in indirect bracket bonding, Concise, and Sondhi. It was found that the addition of CNT had no impact on the SBS or the ARI of the brackets and found that there was no benefit in the addition of CN to orthodontic adhesives [105]. According to Argueta-Figueroa et al. [208] brackets that were directly attached with an orthodontic glue containing CuNPs had a noticeably greater SBS. Turagam and Mudrakola [112] observed a substantial decrease in 0.25 and 0.125 wt% concentrations but no polymerization shrinkage of PMMA resin combined with 0.5 wt% of CNT. Comparing PMMA resins integrated with CN to unmodified PMMA resins, Yeung et al. [209] found reduced polymerization shrinkage in comparison to the integration of 0.25 and 0.125 wt% of CN.

Lewis and Mladsi [210] found enhanced stiffness and TS of PMMA resin incorporating 0.5 wt% of CN. The alternative CN types (multiwalled kinds), incorporating NPs of distinct chemical elements (Cu, Ag, and Au), or integrating them into the composite resin utilized for bonding can be considered. Suo et al. [87] discovered that both modified SWCNTs and MWCNTs formed a stable coating on the dentin surface without affecting SBS. Several studies have found that a CNT suspension could inhibit the activity of *S. mutans* [211–213], while the results by SUO et al. [87] found that the activity of *S. mutans* could be inhibited by SWCNT coating on the dentin surface, but not by MWCNT coating. This indicates that SWCNT coating may be a promising antibacterial material for dental bonding. Alsunbul et al. [106] found that adding 2.5 wt% of CNPs and GNPs to a CA resulted in the 2.5% GNP adhesive showing the best interaction with root dentin and acceptable rheological properties. Compared to the CA, a reduced degree of DC was observed for the 2.5% CNP and 2.5% GNP adhesive [106]. Previous studies have confirmed that NP supplementation in adhesives can reduce its DC [214, 215]. A higher DC of the adhesive allows adequate monomers to be polymerized, preventing of microleakage and secondary caries development. Conversely, a low DC (possibly due to the presence of unreacted monomers within the matrix) can negatively impact the material's biomechanical properties and longevity [216–218].The inclusion of CNPs and GNPs as fillers in the adhesive could enhance their mechanical properties, suggesting promising potential in clinical applications in dentistry.

#### 9.1.3. GIC

Though GIC has multiple advantages but its use in the restorative dentistry has been restricted due to its strength. Various modifications in its composition have been tested earlier to improve its physical and mechanical properties. Recently, incorporation of graphene, CNTs, HA, and bioactive glass into GIC has gained some popularity to manufacture smart dental materials. However, there are varying results, especially when physical, mechanical, and biocompatibility properties are assessed. Piyush et al. [219] compared the compressive strength, microhardness, diametral TS, and biocompatibility of GIC after adding HA, MWCNTs, graphene, and bioactive glass in a 10:1 weight ratio. They found the highest compressive strength, diametral TS, and microhardness of GIC by adding graphene and MWCNTs, with minimum cytotoxicity in the CNT group [219]. Similarly, Foroughi et al. [107] found improved mechanical and bioactivity properties of GIC by adding CNTs/BG. The electrical conductivity also increased after incorporation of CNTs due to the formation of a percolating network of CNTs within the dense matrix [107]. Spinola et al. [111] observed reduced compressive strength and increased TS of GIC after incorporating MWCNTs. In contrast, Hamdy [159] noted the addition of Ag doped MWCNT fillers in GIC improved its compressive strength, surface microhardness, and antimicrobial effect. AlMufareh et al. [110] found improvement in the compressive strength of the GICs by incorporating the SWCNTs over MWCNTs. However, an increase in the surface roughness with no improvement in the nanohardness was noticed by adding CNTs when compared to Ag oxide NPs [110]. Similarly, Goyal and Sharma [109] found improvement of mechanical properties of GIC by adding CNTs; however, the color of the mixture turned black. Therefore, use of this mix was recommended only for posterior teeth. In the current scenario when patients need esthetically pleasing restorations, their use in day-to-day practice needs to be further evaluated [109]. Pani et al. [108 observed better color stability of GIC cement by adding CNTs than AgNPs. This could be due to increased mechanical properties of GIC by reinforcing it with CNTs.

MWCNTs are like a number of tubes nested inside each other, similar to the rings of a tree. It is easy to synthesize, unlike SWCNTs with one layer thick. MWCNTs are long tubes, but the length itself is not the crucial characteristic in many applications. The ratio of the length to the diameter is often more important. For MWCNTs with diameters between 7 and 100 nm, the aspect ratio is typically between 50 and 4000, while single-layer SWCNTs are thinner (0.5 to 2.5 nm) with their aspect ratio up to 10,000. The addition of single walled CNT into GIC may be more effective due to its smaller diameter and higher ratio. Additionally, SWCNT with its single-layer wall is more flexible than MWCNT due to the many concentric layers in the wall of an MWCNT, making it thicker and more rigid.

CNTs enhance the mechanical properties in many ways such as by interlocking carbon-to-carbon covalent bonds, avoiding the boundaries between the crystalline grains, being strong but elastic, and absorbing high amount of force without undergoing the break in the bonds of the atomic lattice. Carbon atoms in NTs can covalently bond to other atoms or molecules, creating a new molecule with customized properties. Bonding an atom or molecule to a NT to change its properties is called functionalization.

Thus, the addition of single or MWCNTs has shown to improve the mechanical and biological properties of GIC. However, method of preparation, diameter, concentration, and surface modification of CNTs can influence the overall outcome. Therefore, a well-designed methodology with detailed characterization of CNTs and assessment methods is needed to validate the effect of CNTs in GIC.

### 9.2. Structural Analysis on Use of CNT for Prosthetic Biomaterials

#### 9.2.1. Denture Base Resins

Turagam and Mudrakola [112] found that PMMA containing varying quantities of CNTs showed decreased polymerization shrinkage. These results are consistent with earlier research by Yeung et al. [209] which revealed that total polymerization shrinkage in PMMA resins was greatest in the absence of NTs as opposed to their presence [220]. The study also revealed that 0.5 wt% CNTs aided in a significant reduction of polymerization shrinkage, which is similar to results of earlier studies by Lewis and Mladsi [210]. Wang et al. [65] conducted their research, they found that the mechanical characteristics of PMMA decreased when 2 wt% of MWCNTs was added to the mixture. This finding is aligned with previous research that has shown that MWCNTs tend to agglomerate when they are processed into polymers. As a potential solution to this issue, the authors proposed the incorporation of surfactants into the PMMA monomer [221, 222].

In the study by Mahmood [113], the IS increased with the addition of 1 wt% CNT, which was also observed in the study by Mars et al. [223] that found CNTs were strong and stable due to the carbon's hexagonal ring arrangement, which reduces segmental motion and increases IS. In Mahmood's [113] study, the results of the transverse strength test after the addition of CNT showed higher values which were consistent with the study by Ayad and Badawi [160]. The observed improvement in transverse strength was attributed to the effective dispersion of NPs, which allows them to occupy the spaces between polymer chains and impede chain movement. This, in turn, enhanced strength and rigidity and was comparable with the findings of Wang et al. [65] and Alwan et al. [224].

A study conducted by Kim et al. [64] to test CNT reinforced PMMA showed a significant reduction in FS and flexural modulus were observed in all CNT groups except 0.25% CNT compared to pure PMMA. Further at 2 wt% concentration, the IS was decreased significantly. These findings were consistent with previous studies that have reported higher contents (>2%) of CNT in PMMA and other biopolymers that could be responsible for poor mechanical properties while up to 0.5%–1% CNT may in fact strengthen the microstructure of composite [65, 225, 226]. CNT–PMMA exhibited antiadhesive effects against all investigated microorganisms, and these effects were enhanced as CNT incorporation was enhanced, consistent with previous research [227, 228]. Studies have shown that phospholipid extraction, membrane cutting, and the breaking of microbe chains (via interconnected filaments) when microbes are directly exposed to CNTs [79, 82, 229]. Thus, it was suggested that the disconnection of sequential microbe chains as confirmed by SEM and staining in this study may be key factors in the antimicrobial adhesion mechanism of CNTs.

CNTs bridged cracks in PMMA samples containing 0.5 wt% MWCNT, increasing their mean FS, according to Ghosh and Shetty [114]. Carbon's spatial hexagonal ring structure in NTs lowers segmental motion, strengthening and stabilizing the mixture. These NPs can fill polymer chain gaps due to their dispersibility, limiting chain movement and increasing stiffness and strength. Carbon atoms are closely connected, making NTs more stable. Compared to the control and MWCNT, 0.5 wt% GO had reduced FS. This may be because graphene sheets agglomerate and behave as micrometer-sized low-surface-area fillers. Agglomerates often form steric barriers that inhibit chemical processes due to the large size of molecular groups. Limiting polymer flow creates solution voids. Void stress concentrations in the matrix increase failure risk (71). Poor filler–matrix interfacial interaction makes polymer composites with balanced strength-toughness difficult to make [114]. One of the main challenges in producing polymer composites with balanced strength-toughness is the inadequate interfacial interaction between the fillers and matrix.

Song et al. [115] synthesized a PMMA/Ter-CNT composite by grafting, which has balanced strength–toughness, self-cleaning, and high thermal conductivity. During tensile extension testing, the PDMS phase in the terpolymer may have concentrated stress in the PMMA matrix, causing internal cavitation in the domains or interfaces. Cavitation may also release energy and toughen. CNT's strong thermal conductivity speeds up heat transmission and improves thermal conductivity, raising the matrix temperature around the fillers during the thermal test. High PDMS content on the composite surface lowered surface free energy, making it hydrophobic and self-cleaning [115].

Mohamad et al. [116] observed that in the case of the MWCNT-containing groups, the improvement in FS was ascribed to the MWCNTs' intrinsic elastic property, which allows them to be bent and distorted without breaking. Because they are filamentous, NTs have also been observed to have a bridging effect. They also have a high degree of stiffness, which raises the elastic modulus. Thus, MWCNT-reinforced 3D printed dentures with improved mechanical properties can help reduce midline denture fractures and promote longer-term wear [116].

Alhotan et al. [88] found that 0.05 wt% Ag-doped CNT and PMMA boosted IS, microhardness, and anticandida action. Since Ag-doped CNT NP fillers attached effectively to the resinous matrix, the enhanced PMMA had better IS. Stress is transferred from the weaker polymer matrix to the robust fillers by this high interfacial interaction, preventing cracks. Hard CNT NPs were equally dispersed over acrylic denture base materials, which may explain the increase in surface microhardness. AgNPs can rupture *Candida albicans*' outer cell membrane and prevent prosthetic colonization. Even though the treated denture base material's anticandidal effect with the Ag-doped CNT was only 2.7 mm, its intimate contact with oral mucosa may alter alveolar mucosa intraorally [88].

According to the study by Swaroop et al. [117], it was found that the mechanical properties such as FS and IS increased significantly by adding 0.5 wt% MWCNTs or 0.25 wt% MWCNT + 0.25 wt% graphene to PMMA monomer (liquid). The findings are aligned with studies conducted by Wang et al. [65] Swami et al. [230], and Ghosh and Shetty. [114]. CNT's spatial hexagonal ring structure inhibits segmental motion, giving the mixture stability and strength. CNTs have a significant dispersibility, which allows them to occupy the spaces between polymer chains to increase strength and stiffness while limiting chain movement. Furthermore, any pressure placed on the polymer matrix is transferred to the NTs, ideally with a strong interfacial bond between PMMA and CNTs, which is the case at concentrations of 0.5% and 1%. Aldwimi et al. [161] found that reinforcing PMMA with HNT and MWCNT makes it more stress resistant. This method boosts PMMA fracture resistance by reducing crack energy and changing NPs from tetragonal to monoclinic. This phase compresses the fracture due to crystal expansion, preventing the break from expanding [118]. This study confirmed Dagdiya et al. [231] findings that adding 8% and 13 wt% aluminum oxide powder to heat-cured PMMA denture base resin considerably improved FS. The treatment of HNTs and MWCNTs may have improved dispersion and interfacial bonding in the PMMA matrix and nanofiller. The composite's FS increased due to improved dispersion and interfacial bonding. Fine nanofillers strengthen the stiffness of a linear macromolecular polymer by filling in the gaps between the chains and limiting segmental movement. The synergistic effect of MWCNTs and HNTs, which strengthen and bond, increased the composite's mechanical properties.

#### 9.2.2. Zirconia

Khan et al. [119] in their study concluded that the adhesion strength of silica coated zirconia to resin cement was significantly increased by the infusion of SWCNTs blended with experimental silanes containing ACPS and BTSE. The reason suggested for this was that the 1.5 nm long NTs may either provide locations for mechanical interlocking at the nanoscale level or strengthen the weak intermolecular interactions of the silane primers. It is possible that the resin composite cement with ZrO_2_ adhered to the NTs more strongly because of their penetration into the pores, perforations, and other irregularities of the ZrO_2_ substrate [119]. Jang et al. [120] tested the mechanical properties of 3YSZ composites containing various concentrations of MWCNT and found that as the concentration of MWCNT increased, the fracture toughness of the zirconia composite increased while its hardness decreased. This may be due to decreased phase transformation of tetragonal zirconia to monoclinic phase subsequent to stress release caused by increased CNT content. This results in a material that has excellent mechanical dependability and great fracture toughness without sacrificing hardness [120].

In a study by Son et al. [121], it was concluded that DRCs blended with any concentration of CNT do not yield high FS and hardness and may not be preferred for reinforcing DRCs. This was due to the presence of pores, as revealed in the SEM analysis of CNT containing DRCs. The increased resin viscosity brought on by adding more CNTs may be the cause of these pores. Due to its higher viscosity, the free space left by the base plate may not get entirely filled and this can cause pores to appear in the printed structure and consequently lower hardness values. da Silva et al. [122] investigated the characteristics of silica-coated CNT-containing ceramic nanocomposite (Y-TZP/MWCNT-SiO_2_) and found acceptable optical properties. The VH and fracture toughness did not show any improvement, while the FS decreased as compared to Y-TZP alone [122]. These findings are not consistent with those of Garmendia et al. [232] which demonstrated high values of hardness and fracture toughness of Y-TZP/MWCNT-SiO_2_ in comparison to a control sample of Y-TZP. This rise was attributed to the impact of toughening mechanisms, like crack-bridging, that are linked to the material's CNT content [232, 233].

### 9.3. Structural Analysis on Use of CNT for Bone Regeneration

Modern tissue engineering aims to create tissue replacement by culturing bone cells on biodegradable or nonbiodegradable synthetic 3D scaffolds or live prostheses. CNTs demonstrated their promising capability as an inert matrix to allow cell proliferation and become functional, normal bone [234, 235]. The highest cell growth was seen at electrically neutral CNTs, whereas the lowest growth observed on AP-MWNTs may be partly due to the detachment of MWNTs into the solution, resulting from reduced attachment of this CNT type to the glass [236]. Plate-shaped crystals seen on SWNTs which are morphologically similar to HA crystals in woven bone, suggest that CNTs provide a suitable substrate for deposition of a mineralized matrix [237].

Gholami et al. [124] observed that HA/MWCNTs-COOH/BSA showed the highest compressive strength. This is because of the presence of the f-CNTs which improves dispersion of MWCNTs in the HA matrix that creates better interfacial bonding strength and easier links to other needs groups modified [124]. At low concentrations, all the developed composites showed no cytotoxic effects on cell proliferation, but as the concentration increases, the cell viability decreases. This is due to the hydrophobic nature of non-f-MWCNTs which causes aggregation and interactions with cells inducing apoptosis. Pulskamp et al. [238] found that iron catalyst residues reduce cell viability, while other research has found that catalyst residues did not affect the materials, as residues remained trapped in the interior of the CNTs. Therefore, if residues played a role in the in vitro results, the cell viability of composites using low purity MWCNTs would be less than the composites that used high purity MWCNTs.

Zhang et al. [239] showed that the MWCNTs which were chemically modified with carboxylic acid groups (–COOH) demonstrated a sharp increase in the cell viability of osteoblasts [240]. Scaffolds used in bone regeneration possess the ability to be broken down by the body, have strong mechanical properties, retain and release growth factors, and are readily available to cells [166]. There is a requirement for a scaffold that is not capable of being broken down by natural processes but has the potential to stimulate the growth of new bone tissue and fill gaps in bone structure. As per the study done by Tanaka et al. [125], the bone formation was more effective using RbBMP-2-loaded MWCNT blocks compared to COL sheets, indicating that rhBMP-2 is retained and released from the former. The scaffold's surface, which is flat at a macroscopic level, but has defects at the nanoscale to microscale, promoted cell growth. As a result, cell attachment happened earlier compared to PET-reinforced COL fibers.

Shimizu et al. [241] discovered that the MWCNT scaffold induces the growth and activity of osteoblast-like cells, leading to the initiation of bone calcification. MWCNTs alone can function as a scaffold for BMP, with their effectiveness in supporting bone formation being comparable to COL sheets, which are regarded as the Au standard for BMP-containing scaffolds [242].

Sanchez et al. [127] observed homogenous dispersion gives nanocomposite films with 5% HA NPs and 0.5% MWCNT enhanced mechanical characteristics. HA NPs (5% and 50%) and 0.5% MWCNTs increased mechanical characteristics. MWCNT agglomerated and formed clusters over 1 wt%. Unequal NT dispersion in the matrix reduces mechanical characteristics [243]. The 5% HA NPs and 0.5% MWCNT CS- HA-MWCNT nanocomposite showed the highest cell viability. 30% and 50% HA NPs films had lower cell survival than 5% CS-HA. Particle clustering reduces scattered HA NPs' concentration-dependent cytotoxicity [244]. MWCNT at 3% weight percent was toxic and reduced cell viability [245]. CNTs promote cell survival at reduced concentrations, biomedical applications should use MWCNT at less than 1% of concentration [246–248]

Silva et al. [128] used 0.25 mg of functionalized SWCNT as the minimum required in CS host scaffolds. High SWCN concentrations can cause nanocracks and mechanical property loss. CS and CNTs produced bone with increased regularity, density, and abundance without inflammatory infiltrates [128]. CS is biocompatible, biodegradable, antibacterial, nonimmunogenic, and nontoxic [249, 250]. NTs in CS increase cellular connections, accelerating bone repair. Scaffolds promote mineralization and bone formation [251]. Munhoz et al. [252] found that COL and CS sponge mineralization does not improve bone tissue repair. However, the experiment was done on rats' calvaria, which is unaffected by muscular tension. By stimulating cell signaling, LLLT enhanced bone volume and transformed mesenchymal stromal cells into osteoblasts [253]. LLLT promotes bone tissue growth and extracellular matrix (ECM) consistency, which aids tissue repair.

The MWCNT–PLA composite scaffolds developed by Zahedah and Dinc [254] showed that they could assist bone tissue heal better. They had a glass transition temperature that was 15% higher, cell adhesion that was 20% better, and mechanical strength that was 25% stronger than pure PLA. Characterization using SEM, DSC, and FTIR validated enhanced thermal stability, structural integrity, and biodegradability, whereas HOB cell investigations demonstrated improved biocompatibility and proliferation. These results show how important MWCNTs are for making high-performance, bioactive bone scaffolds that may be used to heal bone defects [254]. CNT–based membranes provide a viable option for GBR through the integration of mechanical strength, selective osteoblast enhancement, and efficient cellular protection. The lack of CNT diffusion and improved bone regeneration underscore its prospective clinical applicability [167]. The PLA/nHA/CNTs-CHX scaffold successfully merges antibacterial action with osteogenesis, overcoming a key challenge in infected bone repair. While CHX ensures infection control, CNTs maintain osteoinductivity without cytotoxicity. Further in vivo studies are needed to validate clinical potential, but this approach offers a promising strategy for complex bone defects [168].

### 9.4. Structural Analysis on Use of CNT for Tissue Engineering

The techniques used to synthesize CS/HA composites are mostly mechanical mixing [44], coprecipitation and an alternate soaking process. However, the distribution of inorganic particles within the organic matrices is not uniform which causes poor mechanical strength, hence, their applications are limited [255]. It was shown that unique morphology and ultrafine HA are distributed evenly in the organic template via in situ precipitation method, hence, promoting high-affinity nucleation and growth of HA in polymer hydrogel [256]. CS/HA composites show favorable degradation with excellent biocompatibility, great tissue regenerative efficacy, and osteoconductivity [257].

Ignat et al. [130] in their study, observed that the total porosity and structural thickness of the membrane reached their maximum at 0.5 wt% GO–CNT concentration. GO interacts better with proteins, hence, providing more adhesion in cell attachment [130]. CNT has a great ability to bind with proteins of the ECM which improves cell attachment and cell distribution [258]. GO also accelerates adipogenesis of MSCs, which is shown by the increased expression of adipogenic markers in tonsil-derived MSCs when seeded in a GO-enriched hydrogel than on a hydrogel without GO [259]. CNTs improve cellular spreading via mechanotransduction, which affects the levels of cell differentiation such as chondrogenesis, neurogenesis, and osteogenesis. The increased osteogenic differentiation on CNT films is thought to be induced by the material roughness which stimulates the cell spreading and the reinforcement of the actin filaments [260].

Fathy et al. [131] in their study, observed that a fall in the stress–strain curve at high stress region. This may be due to the chemical attraction of the composite phase of stiff part f-CNT and tough part of CS polymer, and large areas of osteoconductive HA which favor cell adhesion and proliferation [131]. Good dispersion of CS/f-CNTs in the pores and on the surface of cuttlefish bone enhanced cell interaction which led to low cell viability as compared to groups of agglomerated NTs. This may be attributed to the lack of aggregated MWCNTs that could otherwise enter the cytoplasm and nucleus, leading to cell apoptosis [261, 262]. The authors suggested future research investigating HA/CS/f-CNT scaffolds in vivo to evaluate the cellular proliferation and differentiation of the scaffold.

The addition of small amount of CNTs into various types of scaffolds improves the mechanical properties of the scaffolds [263, 264]. As per findings of Farshidfar et al. [132], the COL–MWCNTs 1%-CUR 10% composite scaffolds exhibited excellent surface wettability and significant decreased porosity. The excellent wettability was contributed by the MWCNTs 1% due to the presence of the functional groups of MWCNTs (COOH) that reduced the static water contact angle (WCA) whereas 10% CUR affected minimally on the WCA value [132]. CNT exhibits significant potential in tissue engineering owing to their diverse characteristics [265]. Nonetheless, obstacles such as enduring biocompatibility and therapeutic applications persist. Subsequent investigations ought to concentrate on enhancing these materials for secure and effective regeneration therapies [266].

### 9.5. Structural Analysis on Use of CNT for Periodontal (Gum Care) Treatment

Mei et al. [133] observed that uniform dispersion of HA NP in the membrane forms a three-dimensional porous structure. HA NP also slows down the degradation rate as the alkaline particles prevent the entry of water. In the cell counting and MTT assay, the PLLA/MWNTs/HA composite membrane showed three times as many PDLCs compared to the initial seeding cells. The PLLA/MWNTs/HA membrane was also one-third larger than the PLLA/HA membrane or control group at Day 7 of culture. From Day 1 to dDay 7 of culture, the cell number of GECs in the PLLA/MWNTs/HA group was always lower than in the PLLA/HA and control groups. The positive interaction between the novel material with PDLCs and the negative interaction with GECs may be due to higher energy and the different surface of the material which may increase osteoblast adhesion and decrease adhesion of its competitive cells [133, 267].

To create a scaffold for tissue regeneration, the viscosity of the polymer is a crucial factor during electrospinning that affects the diameter of the scaffold pores [268]. As per Zarei et al. [135], PHB/1%CNTs scaffold formed more HAE crystals than pure PHB, which is highly potential for PDL regeneration. This is due to the increased functional group of CNTs (COOH) which reduced WCA in the scaffold, resulting in the hydrophobic nature of PHB that prevents water absorption [135]. f-CNTs in the PHB scaffold increased angiogenesis, reduced inflammatory cells, and improved tissue compatibility than pure PHB. Biocompatibility of the scaffold was improved with the addition of CNTs in small amount [247].

### 9.6. Structural Analysis on Use of CNT for Endodontic Treatment

As per observations by Sanaee et al. [136] the compressive and FS of MWCNTs reinforced MTA was improved in 0.25, 0.50, and 0.75 wt% MWCNTs specimen groups. The superior bonding strength between CNT and cement matrix is because of the interfacial interactions between CNTs and the hydrations of cement [269]. Uniform dispersion of CNTs improved the mechanical properties of the cement [270]. However, the flexural and compressive strength reduced in MTA containing 1.0 wt% MWCNTs are due to the non-uniform dispersion of MWCNTs as a higher amount of MWCNTS being utilized. Radiopacity of MTA reduces as the milling time increases. This is due to the ceramic beads and zirconia of MTA colliding with each other during milling, leading to crystallinity loss in zirconia particles and an increase in the proportion of MTA/water [271].

BioRoot root canal sealer is popular for its biocompatibility, antibacterial properties, and osteogenic potential. Nanomaterials in 1 wt% composites accelerate cement hydration, improving properties. A 2 wt% composites have longer initial setting times. The powder to water ratio significantly impacts the physicochemical properties of calcium silicate sealers. Shorter setting times reduce solubility due to less contact with body fluid. Bioroot/1wt% CNT and TC have reduced solubility due to water absorption. Bioceramic RCS increases solubility but may increase apical leakage risk. A 1 wt% composites have higher pH, with Bioroot/1 wt% BN and Bioroot/1 wt% TC having the highest alkaline pH. Further investigations are required to study the chemical analysis of the nanomaterial components and a more effective method to distribute the nanomaterials more homogenously in calcium silicate sealers [137].

Another study by Baghdadi et al. [138] found that Bioroot/MWCNT and Bioroot/TC composites showed maximum strength, except for BN-reinforced BioRoot RCS. BN particles cause cleavage fractures due to their large size. MWCNTs and TC enhance compressive strength by bridging cracks and forming crystals within the cementitious matrix. However, adding 2 wt% nanomaterials to BioRoot RCS results in non-homogenous dispersion, entanglement, and higher porosity, reducing compressive strength [138]. CNTs and AgNPs can be used as antimicrobial agents in resin matrix materials, acting as a delivery platform for CHX. CHX inhibits matrix metalloproteinase (MMPs), while AgNPs eliminate biofilms. CNTs/AgNPs/CHX 2% particles showed superior antimicrobial properties after setting, suggesting they could act as an active ingredient reservoir. The modified sealer adapted well to root canal walls without affecting structural or thermal behavior. The NPs' dispersion increased the formation of small cleavage planes [139].

### 9.7. Structural Analysis on Use of CNT for Dental Implants

Esmaili et al. [145] in their study observed that addition of Ti-Ta_2_C/MWCNT multilayer increased the surface hardness. The Ta_2_C/MWCNT coating layer could prevent failure and loosening of implants due to its ability to increase fracture toughness, elastic modulus, and resistance to plastic deformation. Ti-Ta_2_C/MWCNT samples exhibited higher corrosion resistance due to lower volume loss [145]. Malekahmadi et al. [146] demonstrated in their study that high thermal conductivity of CNT particles led to higher thermal conductivity of hybrid nanofluids of CNT + HA. While the thermal conductivity of mono-nanofluid of HA decreased due to the insulation of HA particles [146]. Vijay et al. [194] in their review mentioned that the versatility of nanomaterials, especially carbon nanomaterials for biomimetic dental implant modification that can address problems like infections, delayed wound healing, and osseointegration, produce long-lasting dental implants, and help develop customized dental therapy for patients with delayed wound healing who are diabetic or nondiabetic [272]. Sivaraj et al. [147] in their study mentioned that the presence of Cu ions in the composite impart a satisfactory antimicrobial activity against *E. coli* as revealed by a clear fragmented DNA ladder. An implant coated with a hybrid nanocomposite that exhibits enhanced osteogenic differentiation and higher cytocompatibility will be a more suitable option for bone tissue engineering applications. This new Cu-HAE/f-MWCNT hybrid is a strong contender for dental and orthopedic implant uses [147].

Research investigations indicated that MWCNT-enriched coatings markedly boosted dental implant performance. Logesh et al. [196] demonstrated that hydroxylated MWCNTs within HAE coatings improved surface characteristics, antibacterial efficacy, and osteoblast viability in TiNbZr implants. Logesh and Choe [197] demonstrated that MWCNT–Ag/HA coatings on Ti-6Al-4V implants enhanced corrosion resistance (3.25 × 10^15^ Ω cm^2^), mechanical strength, and antibacterial properties. Collectively, these results validate the potential of MWCNTs in the advancement of high-performance, bioactive, and resilient dental implant coatings. CNT-doped implants enhance cell adhesion, diminish infection risks, and lower the necessity for surgical revisions, rendering them advantageous for implant applications [273].

The incorporation of CNTs in dental implants has markedly progressed nanodentistry, providing improved mechanical strength, corrosion resistance, and bioactivity to titanium surfaces. As a crucial nanomaterial, CNTs enhance longevity and functionality, facilitate osseointegration, and diminish therapeutic invasiveness. Their integration in nanomodified coatings exhibits exceptional outcomes in both in vitro and in vivo research, facilitating the development of durable, high-performance dental implants, even for patients with complex circumstances. Future study seeks to enhance their clinical applicability for wider therapeutic use [274, 275].

### 9.8. Structural Analysis on Use of CNT Reinforced Composites

CNTs are transforming dentistry due to their remarkable strength and versatility, functioning as reinforcements for dental material. CNT-reinforced composites have significant potential in dentistry, improving mechanical strength, biocompatibility, and functional efficacy. They enhance load-bearing capacity, wear resistance, and adhesion strength in materials like GIC, resins, and Mg-based implants. Surface functionalization improves dispersion, compatibility, and osseointegration. Grafting bioactive polymers improves adhesion and mineralization, facilitating applications in scaffolds and drug delivery systems. Researchers have integrated CNTs and TiO_2_ NTs with SiO_2_-treated organosilane bonding agents to augment the FS of resin-based composites. This amalgamation enhances the mechanical efficacy of tooth restorative substances [276]. The MWCNTs nanocomposites that improves the mechanical properties of dental resins, including TS and fracture resistance [277].

CNTs exhibit exceptional TS, exceeding 50 GPa, and possess a high Young's modulus of up to 1 TPa. Consequently, they are very appropriate for applications in reinforcing polymers, metals, and ceramics within composites [278]. They function as effective strengthening agents in polymer composites; even small quantities can markedly enhance the mechanical characteristics of polymers. As the need for polymer composites expands across several industries, there is an expectation for CNT composites to develop in increasingly varied directions [279]. Future research will concentrate on hybrid methodologies to surmount these obstacles and fully use the potential of CNTs in enhancing diagnostic and therapeutic applications in dentistry.

Research underscores the potential of CNTs in augmenting biomaterials for dental and osseous regeneration. Al-Harbi et al. [202] established that SWCNTs enhance the mechanical and thermal properties of dental membranes without inducing cytotoxicity, while they do not exhibit antibacterial activities. Zhao et al. [203] shown that MWCNT-reinforced PCL/BC scaffolds possess significant strength and osteogenic potential, efficiently healing mandibular lesions. SWCNTs improve biocompatibility in dental applications, whereas MWCNTs are superior for structural reinforcement in bone regeneration. Also MWCNT composites exhibited potential for bone regeneration, additional refinement of their composition and sustained biological efficacy is required to enhance clinical applicability [204]. Collectively, these findings highlight the adaptability of CNTs in enhancing biomaterials for various therapeutic requirements, balancing mechanical efficacy and biological safety [203].

### 9.9. Critical Appraisal of Novel Developments and Unique Contributions of This Review on CNTs

The presented manuscript presents a meaningful contribution to the current body of knowledge on CNTs in dentistry by offering a well-rounded and up-to-date review of their dual function, enhancing dental materials and serving therapeutic purposes. While most past studies tend to examine CNTs from a single angle such as improving strength or offering antimicrobial effects, this review brings together a broader, multidisciplinary understanding of how CNTs can enhance both the physical and biological performance of dental materials. Key highlights form this review are stated below.

#### 9.9.1. Dual Functionality

Unlike previous reviews that mainly emphasize mechanical upgrades—like better FS or fracture resistance—this manuscript shows how CNTs can do more than one job. For example, they can reduce polymerization shrinkage and prevent bacterial growth in restorative materials [64, 99].

#### 9.9.2. Biocompatibility Evaluation

While concerns about CNT toxicity are well-known in the environmental and materials science fields, this review takes a dental-specific approach. It explores how surface modifications, such as carboxylation or PEGylation, can improve compatibility with oral tissues without compromising performance [42].

#### 9.9.3. Exploration of Emerging Therapies

The review looks beyond the usual material enhancements to highlight newer applications like CNTs as drug carriers for localized cancer treatment [103] or coatings to improve implant integration with bone [187]. These are exciting developments that have not been widely covered in dental literature.

#### 9.9.4. Clinical Implications

Many previous works overlook the practical challenges of translating lab results to clinical use. This review addresses real-world issues like color changes in GICs, long-term stability of CNT-reinforced prosthetics, and proposes realistic solutions such as optimizing filler content and using hybrid nanomaterials.

Overall, by weaving together experimental insights, clinical perspectives, and future opportunities, this review does not just summarize existing knowledge but it offers a strategic guide for advancing CNT use in dentistry. This broad, integrative approach clearly sets it apart from narrower and single-topic reviews.

## 10. Toxicity of CNTs

Understanding the toxicity of CNTs, their accumulation in the body, their detrimental consequences, and potential ways for mitigating toxicity is essential for the advancement of nanomedicine [280]. CNTs demonstrate toxicity in dental biomaterials via many principal routes, predominantly associated with their distinctive physicochemical characteristics. These pathways encompass oxidative stress, inflammatory reactions, and genotoxicity, which may result in numerous detrimental health outcomes. Comprehending these routes is essential for evaluating the safety of CNTs in dental applications. The main concern with employing CNTs is their possible cytotoxicity to healthy tissues. Due to their high aspect ratio, CNTs generate ROS and free radicals, which touch biological membranes and absorb and transport poisons [281]. These mechanisms include inflammation, oxidative stress, and DNA destruction, which may be useful for eliminating tumor tissue but hazardous to normal cells [282]. CNT toxicity depends on aspects such as structure, surface shape, concentration, delivery route, interaction with other substances, and biological susceptibility. They can provoke oxidative stress by producing ROS, which harm cellular constituents. This stress is affected by parameters including CNT size, length, and aggregation, which influence their interaction with biological systems [283].

Recent investigations on the integration of CNTs into dental materials have revealed both significant improvements and considerable biocompatibility issues. Spinola et al. [111] assessed MWCNTs functionalized through acid oxidation (H_2_SO_4_/HNO_3_) at 1 wt% in both conventional and high-viscosity GICs. The addition enhanced diametral TS, but markedly diminished compressive strength and impaired handling qualities, suggesting restricted clinical use at this concentration [111]. Foroughi et al. [107] augmented GIC using a nanocomposite of 1 wt% MWCNTs and 10 wt% bioactive glass, which improved mechanical strength; nevertheless, fibroblast cell culture experiments indicated reduced cell proliferation, hence, raising concerns over cytotoxicity. Conversely, Hamdy et al. [159] illustrated that the integration of an ultralow dose (0.01 wt%) of Ag-doped MWCNTs into GICs resulted in a significant enhancement in compressive strength (from 105 to 172.7 MPa), coupled with antimicrobial effectiveness against *Streptococcus mutans*, without eliciting cytotoxicity in L929 fibroblast assays. Goyal and Sharma [109] evaluated several nanofillers-CNTs, GO, HAE, and bioactive glass at a 1:10 additive-to-GIC ratio, reporting that the CNT group demonstrated the lowest cell viability (~58%), while graphene and HAE displayed superior biocompatibility (~80%–90%).

Although CNTs present potential applications in dental biomaterials, their toxicity poses considerable issues. Continuous research is crucial to create safer alternatives and reduce the dangers linked to their application in healthcare environments.

## 11. Future Considerations and Research Gaps

CNT-containing dental materials are being researched for mechanical, physical, and chemical improvements. This includes improving GIC and resin composites' toughness, resilience, and appearance. CNTs can improve dental procedures due to their adjustable features. Understanding CNT compatibility and safety over time is crucial for incorporating them into dental materials. Future studies must examine CNTs' interactions with biological tissues to prevent long-term harm.

CNTs could be used to develop enhanced dental drug delivery systems for caries and periodontal infections. CNT functionalization should be studied to improve medicinal agent delivery. Future research on CNTs in tissue engineering, particularly in scaffolds that imitate the ECM, seems encouraging. This might give rise to novel oral tissue regeneration and repair methods. CNTs and other nanomaterials may synergistically improve dental materials. Future research should combine CNTs with other NPs to improve antibacterial and mechanical capabilities.

To address research gaps, CNT-enhanced dental materials need uniform mechanical and biological testing techniques. This will aid study comparisons and clinical application recommendations. Long-term clinical trials are needed to evaluate CNT function and safety in real-world dentistry settings. CNT–based materials' durability and efficacy in therapeutic settings should be studied. Research should address ethical, safety, and regulatory issues related to CNTs in dentistry to ensure novel materials meet safety criteria before clinical usage. Understanding public opinion and acceptability of nanotechnology in dentistry is crucial. Consumer perceptions concerning CNTs and their use in dentistry should be studied to improve decision-making and patient trust. CNTs in dental materials must be compared to existing materials for cost-effectiveness.

## 12. Conclusion

CNTs have demonstrated remarkable potential in the field of dentistry due to their exceptional mechanical strength, high surface area, electrical conductivity, and antimicrobial properties. These unique characteristics make them attractive for various dental applications, including reinforcing dental composites, enhancing the durability of implants, promoting bone regeneration, and serving as carriers for targeted drug delivery. Researchers are exploring ways to reduce CNT toxicity, such as surface functionalization and encapsulation in biocompatible materials. Further research is needed to understand CNT toxicology and set safe usage limits. CNT–based dental materials should be used cautiously, prioritizing patient safety.

## Figures and Tables

**Table 1:** Biological properties of CNT-incorporated materials (cellular assays).

Author	Material type/CNT type	CNT type and concentration	Cell line	Type of assay	Biological response	Reference
Kim et al.	PMMA denture base	COOH-MWCNTs, 1 wt%	Human oral keratinocytes	Metabolic adhesion assays	35%–95% reduction in oral pathogen adhesion; no cytotoxicity	[64]
Wang et al.	Acrylic denture base	MWCNT, 0.5–1 wt%	L929 fibroblasts	Fatigue, flexural and MTT assay	↑ Flexural strength; no significant cytotoxicity	[65]
Mattioli-Belmonte et al.	PCL–MWCNT scaffold	MWCNT ~0.3 wt%	hDPSCs	Proliferation assay	Not cytotoxic; supports stem cell proliferation and osteogenesis	[66]
Peñaranda-Armbrecht et al.	PCL/MWCNT scaffold	MWCNT, 5 wt%	L929 fibroblasts	MTT assay	5% formulation supports ~90% cell viability	[67]
Montanheiro et al.	PHBV–CNT composite	GABA- and COOH-MWCNT	Fibroblasts (unspecified)	24 h MTT assay	≥70% viability at 24 h; mild antimicrobial effect	[68]
Harrison and Atala	CNT–polymer composite	MWCNTs (0.1–1 wt%) in PLGA	Human osteoblasts (hFOB)	AlamarBlue viability assay	Enhanced cell adhesion and proliferation; no cytotoxicity at ≤0.5 wt%	[69]

**Table 2:** Comparative characteristics of carbon nanotube-incorporated materials in dental applications.

Characteristics	Characteristics of conventional dental material	Characteristics of CNT-incorporated dental material	Impact on dental biomaterial	Reference
Mechanical strength	70–110 MPa (flexural)	Up to 150 MPa (flexural)	Better fracture resistance	[70]
Fracture toughness	0.7–1.2 MPa m^1/2^	1.3–1.8 MPa m^1/2^	Improved longevity in stress-bearing areas	[71]
Elastic modulus	3–6 GPa	6–12 GPa	Matches dentin properties	[72]
Thermal conductivity	Low	0.5–1.0 W/m K	Reduces thermal shock	[41]
Electrical conductivity	Insulator	0.1–10 S/m	Enables biosensing applications	[73]
Wear resistance	Moderate	Superior	Ideal for posterior restorations	[74]

**Table 3:** Antimicrobial properties of CNT-incorporated dental materials.

Material/application	CNT enhancement action	Effect on secondary caries and longevity	Reference
Resin composites with CNTs	Contact-killing action disrupts *S. mutans* membranes	Inhibits biofilm formation on restoration margins and prevents recurrent caries	[86]
CNT-coated dentin bonding systems	SWCNTs on dentin surface offer strong antibacterial effect vs. *S. mutans* without reducing bond strength	Reduces microleakage and secondary caries risk due to antibacterial seal	[87]
Silver-doped CNT in PMMA denture base	Ag-CNTs exhibit clear inhibition zones vs. *C. albicans* and bacteria	Limits fungal/bacterial colonization → extends denture lifespan	[88]
CNT-reinforced GICs	Showed significant reduction in *S. mutans* adhesion due to CNT-mediated oxidative stress on bacteria	Enhanced durability and antibacterial properties, reducing recurrent caries around GIC restorations	[89]
CNT incorporated in PMMA composites	Exhibited significant drug-free antimicrobial properties, reducing microbial adhesion by 35%–95% against S.aureus, S.mutans, and *C. albicans*	CNT–PMMA composites as promising candidates for preventing microbial-induced complications in dental applications	[64]

**Table 4:** CNT applications for dental biomaterials.

Author (year)	Type of CNT	Objective	Method of preparation for CNT	Composite of CNT with any other NP or surface modification of CNT (example- addition of nano-SiO_2_ onto the oxidized surface of SWCNTs	Concentration (eg: 0.006) or percentage (eg- 5%w/vol) or quantity (mg/g) of CNT and comparative group	Diameter of CNT	Sample size	Tests used for investigation	Outcome	Reference
Composite resins										

Zhang et al. (2008)	Single-walled carbon nanotubes	To improve the application of SWCNTs in dental resin-based composites by depositing a thin shell of nano-SiO_2_ onto the oxidized surface of SWCNTs by using a thin adlayer of 3-aminopropyltriethoxysilane, followed by modification of the nano-SiO_2_ layer by using another organosilane with allyl-terminated functional groups	Blending manually SWCNTs, silicon dioxide and allyltriethoxysilane in urethane dimethacrylate (UEDMA) monomer	Surface modification by nano-SiO_2_	0.1 wt%	<10 nm	NA	Transmission electron microscopy (TEM), FTIR, flexural strength (FS)	Addition of modified SWCNTs improves the flexural strength of dental resin based composites	[99]

Zheng et al. (2019)	Single walled/multiple walled	To evaluate the mechanical properties of dental resin matrix by incorporating MWCNTs modified by different amount of silane coupling agents named 3- (methacryloxy)propyltrimethoxysilane (γ -MPS)	Mechanical blending, mx-CNTs or unmodified CNTs-OH (p-CNTs) are added into Bis-GMA/TEGDMA	Bioactive glass, titanium nanotube, etc.. or surface modification by SiO_2_	0.006 or 5%wt/vol or 5 mg/mL	<20 μm	*N* = 10	TEM, double bond conversion (DC), flexural strength (FS), volumetric polymerization shrinkage (VPS), composite film thickness (CRFT) and modulus (FM)	Incorporation of 0.025 wt% of CNTs modified by 1.0 wt% of silane coupling agents significantly increases the mechanical properties of dental resins	[100]

Borges et al. (2020)	MWCNTs	To evaluate the mechanical properties of composite resin filled with nylon-6 nanofibers (N6) with MWCNTs (N6-MWCNTs)	Electrospinning technique CNTs are purified by hydrochloric acid, washed with water, functionalization in cold plasma chamber then mixed with a magnetic stirrer for 24 hr in 1,1,1,3,3,3-hexafluoro- 2-propanol (HFP) solution	NA	2.5%, 5%, 10% and 20%	<10 μm	NA	Scanning electron microscopy (SEM), volumetric polymerization shrinkage (VPS), film thickness (CRFT), flexural strength (FS) and elastic (*E*) modulus	10% and 20%. N6-MWCNT particles in composite resin based material produces the least polymerization shrinkage; however, 2.5% and 5% show higher suitable flexural strength with reduced film thickness	[101]

Pennisi et al. (2020)	SWCNTs	To evaluate the flexural resistance of indirect composite resin after incorporation of SWCNTs	Mechanical blending by spatulation	Surface modification by SiO_2_	SWCNTs 0.1%, 0.2%, and 0.3% (*w*/*w*) SWCNTs-SiO_2_ 0.1% (*w*/*w*).	<10 μm	*N* = 10	Transmission electron microscopy (TEM), flexural test	Addition of SWCNTs does not improve the flexural strength of indirect composite resin. However, SWCNTs/SiO_2_ significantly improves the flexure resistance. Silanization process improves the strength proprieties of SWCNTs	[102]

Zhouyi et al. (2023)	MWCNTs	To evaluate the mechanical properties of CNTs reinforced composites and role of CNT in resistance to crack propagation	Floating catalyst chemical vapor deposition (FCCVD) method	NA	NA	NA	NA	Compressive strength using electronic universal testing machine	Addition of CNT in composite reins significantly increases the compressive strength specially under quasistatic conditions and also reduces the crack propagation	[103]

Dental adhesives										

Akasaka et al. (2008)	MWCNTs	To study effects of CNT coating on the surfaces of tooth slices and how CNT coating affects the tensile bond strength of dentin adhesives	Arc-discharge method	NA	NA	200 nm	–	Scanning electron microscopy	HAp nucleation and protection against dental bacteria, without reducing the composite strength of restorative materials	[104]

Marchi et al. (2017)	Single-walled CN	To evaluate the effects produced by CNT added to two adhesives used for indirect bonding	A 10% solution of sodium dodecyl sulfate was prepared as a surfactant to stabilize CN in water and to promote bonding to the monomers of the Sondhi rapid set adhesive base pastes and Concise single-walled CN were added to the solution to obtain a concentration of 1 mg/mL. Homogenization was achieved by ultrasound and added to adhesive base paste to obtain 0.1%, 0.5% and 1% CN concentrations	NA	CN at 0.5%, 0.25%, and 0.05% in Sondhi and Concise adhesives	0.7–1.1 nm	160	Shear bond strength (SBS) using mechanical testing machine and adhesive remnant index (ARI)	The addition of CNT to the Concise and Sondhi adhesives, at the concentrations used, did not improve SBS or the amount of adhesive remnant remaining on enamel (ARI) following bracket removal	[105]

Suo et al. (2018)	SWCNT and MWCNT	To develop CNT coatings for the dentin surface and investigate the bonding strength and the in vitro antibacterial properties of CNT coated dentin	Calcination, acid treatment, and carboxylation used to fabricate CNTs with good dispersion	NA	NA	SWCNT- 1–2 nm MWCNT- <8 nm	60	Optical density using a UV–vis spectrophotometer, FTIR spectroscopy, scanning electron microscopy, transmission electron microscopy	SWCNT coating and MWCNT coating did not affect the immediate shear strength of the dentin bond. SWCNT coating may be a potential antibacterial material when applied to dental bonding	[87]

Alsunbul et al. (2023)	Commercially available CNPs	The study aimed at including 2.5 wt% of CNPs and GNPs in a control adhesive (CA) and then investigate the effect of this inclusion on their mechanical properties and its adhesion to root dentin	Commercially available CNPs	NA	2.5 wt% CNPs in adhesive	NA	45	Scanning electron microscopy and energy dispersive X-ray (SEM-EDX) mapping, push-out bond strength (PBS), rheological properties, FTIR and degree of conversion (DC) investigation, failure type analysis using stereomicroscopy	Both NPs reinforced the adhesive's mechanical properties and their supplementation with adhesives may be of practical significance in dentin bonding	[106]

GIC										

Foroughi et al. (2016)	MWCNTs	To evaluate the effect of CNTs/bioglass (BG) on mechanical and bioactivity properties of glass ionomer cement	Using fusion method: GIC, CNTs and bioactive glass	CNTs/bioactive glass	1 and 2 wt%	Internal diameter 3–5 nm; external diameter of 5- 15 nm	NA	Compressive strength test erosion test, cell culture and analysis, cell viability test (MTT assay)	CNT can increase the compressive strength and resistance to erosion due to its wide distribution. The electrical conductivity of BG also increased after incorporation of CNTs	[107]

Pani et al. (2020)	NA	To compare the staining characteristics of a GIC reinforced by CNTs and silver NPs	Using mixing method	NA	0.03 wt%	NA	*N* = 20	Color stability at 1, 24 h, and 1 week spectrophotometer	CNT reinforced GIC shows better color stability than silver NP reinforced GIC	[108]

Goyal and Sharma (2021)	MWCNTs	To evaluate the effect of CNTs on mechanical properties of glass ionomer cement	Using mixing method	NA	2 wt%	NA	NA	XRD scanning electron microscopy, FTIR, TGA, DSC, setting time, swelling and solubility, compressive strength, hardness, wear	Addition of CNTs improved the mechanical properties of GICs drastically	[109]

AlMufareh et al. (2021)	SWCNTs and MWCNTs	To synthesize and characterize CNTs and silver oxide NPs based GICs and assess their mechanical properties	Using mixing method	NA	0.03 wt%	NA	*N* = 8	Surface roughness (3D optical noncontact surface profiler), nanohardness (nanomechanical tester), compressive strength (universal testing machine)	Incorporation of CNTs improved the compressive strength of the GICs without improving the nanohardness. However, surface roughness also increased but well below threshold	[110]

Spinola et al. (2021)	MWCNTs	To evaluate the effect of MWCNTs incorporation on the compressive and diametral tensile strengths of glass ionomer cements	Using mixing method	NA	1 wt%	25 nm	*N* = 20	Compressive diametral tensile strengths universal testing (universal testing machine)	MWCNTs incorporation reduced the compressive strength and increased the tensile strength of GIC regardless of their viscosity	[111]

Denture base resins										

Turagam and Mudrakola (2013)	MWCNTs	To compare the polymerization shrinkage of PMMA with and without microadditions of CNTs	Commercially available MWCNT powder	NA	0.5 wt%, 0.25 wt%, 0.125 wt% MWCNT in MMA monomer	20–40 nm	*n* = 10	Traveling microscope	Microadditions of CNTs in PMMA especially 0.5 wt% CNT can produce composites with significantly reduced polymerization shrinkage	[112]

Wang et al. (2014)	MWCNTs	To examine how the mechanical properties of PMMA denture base material were affected by MWCNT reinforcement	Commercially available MWCNT	MWCNT–PMMA composite	0.5 wt%, 1 wt%, and 2 wt% of MWCNT	NA	*n* = 7	3-point-bending test for flexural strength, 4-point bending test for fatigue resistance, SEM analysis	MWCNT–PMMA composite resins have better flexural strength and resilience at 0.5 and 1 wt% of MWCNT and a lower fatigue strength	[65]

Mahmood (2015)	NA	To use different concentrations of CNT to improve the mechanical and physical characteristics of a high impact denture resin	Commercially available CNT	CNT added to 10 mL monomer using probe sonication apparatus and then mixed with 21 mg PMMA powder followed by heat polymerization to obtain CNT reinforced PMMA	0%, 0.5%, 1%, and 1.5% by weight of CNT	NA	NA	Impact test, transverse strength using UTM, roughness test by profilometer, shore hardness test	Impact and transverse strengths considerably increased in the presence of 1wt% CNT, while hardness and roughness decreased	[113]

Kim et al. (2019)	MWCNTs	To study the effect of incorporating carboxylated MWCNTs into PMMA on its drug-free antimicrobial adhesion properties	By mixing CNT's in a 1:1 vol% H_2_SO_4_/HNO_3_ aqueous solution followed by filtration and drying	Carboxylated MWCNT	0.25, 0.5, 1.0 and 2.0 wt% CNT	15–20 nm	*n* = 8	Scanning electron microscopy (SEM), 3-point bending test using UTM, Charpy impact test, microbial adhesion test, FUN-1 staining, cytotoxicity test	Because of its remarkable decrease in microbial adherence and sustained mechanical qualities, 1% CNT was determined to be the ideal concentration for usage as an addition to PMMA	[64]

Ghosh and Shetty (2020)	Multiple wall CNT (MWCNT)	To compare the FS of (PMMA) modified by fillers such as graphene oxide (GO) and MWCNT	Commercially available MWCNT powder	NA	0.5 g of MWCNT and 0.5 g of GO in 106 ml of MMA monomer liquid	16–20 nm	*n* = 20	3 point bending test using UTM	MWCNT added at 0.5 wt% to PMMA is a simple, efficient, and cost-effective way to increase its flexural strength	[114]

Song et al. (2021)	Multiple wall CNT (MWCNT)	To create PMMA/Ter-CNT composite with varying concentration of Ter/CNT and evaluate for strength toughness, thermal conductivity and self cleansing properties	Terpolymer grafted MWCNT (Ter/CNT)	PMMA/Ter-CNT composites prepared by solution blending	PMMA/Ter-CNT composite containing 0.5, 1 and 2 vol% of Ter/CNT	10–20 nm	NA	Atomic force microscopic (AFM) analysis, thermal conductivity, contact angle measurements, tensile tests	PMMA/Ter-CNT composite exhibited balanced strength-toughness, good self-cleaning ability and high thermal conductivity	[115]

Mohamad et al. (2023)	Multiple wall CNT (MWCNT)	To evaluate and compare the flexural strength (FS), impact strength (IS), surface roughness (*R*_a_), and elastic modulus of 3D printed denture resin reinforced with 0.25 and 0.5 wt% silane coated MWCNTs and nanoglass particles alone and in combination	MWCNT were functionalized using nitric and sulfuric acids at a 1:3 (*v*/*v*) ratio	MWCNTs were silane coated using APTES and allowed to dry for up to 4 h at 120°C in a muffle furnace	Printable resin modified with 0.25 and 0.5 wt% MWCNTs and nanoglass	45 nm	330	Thermocycling (600 cycles), 3-point bend test under UTM, elastic modulus, Charpy impact test, SEM analysis, surface roughness	3D printed resin's FS, elastic modulus, and IS all improved as a result of the addition of nanoglass and MWCNTs	[116]

Alhotan et al. (2023)	Multiple wall CNT (MWCNT)	To study the impact strength, microhardness, and anticandida activity of Ag-doped CNTs added to heat-cured acrylic denture bases	Commercially available MWCNT powder	MWCNT doped with Ag	0.05 wt% Ag-doped CNT powder blended with 99.5 wt% heat polymerized PMMA powder	NA	60	SEM analysis, energy-dispersive X-ray spectroscopy (EDX), Charpy impact test, digital Vickers hardness test, agar diffusion test with *Candida albicans*	The addition of 0.05 wt% Ag-doped CNT NPs to PMMA resulted in higher impact strength, surface microhardness, and enhanced anticandida activity compared with the control group (PMMA)	[88]

Swaroop et al. (2023)	Multiple wall CNT (MWCNT)	To evaluate and compare the flexural and impact strength of heat-polymerized PMMA (polymer and monomer) reinforced with graphene and MWCNTs and graphene + MWCNTs	Commercially available MWCNT powder	NA	0.5 wt% of MWCNT added to either PMMA polymer or PMMA monomer under ultrasonic mixing	NA	80	3-point bending test, Izod impact test	The best nanofiller to use for heat-cure PMMA reinforcement was 0.5% by weight of MWCNT, as it demonstrated the highest FS and IS when included in the monomer	[117]

Aldwimi et al. (2023)	Multiple wall CNT (MWCNT)	To evaluate and compare the effects of utilizing two treated nanotube fillers such as halloysite nanotubes (HNTs) and MWCNT on mechanical properties of PMMA	Commercially available MWCNT powder	10 wt% of silane coupling agent used to functionalize the surface of HNT/MWCNT nanocomposite	Mixture of 0.25, 0.50, 0.75, 1.0, 1.5, 2.5 wt% of MWCNT and 4.75, 4.5, 4.25, 4.0, 3.5, 2.5 wt% of HNT, respectively (total = 5 wt%) added to MMA monomer liquid	10–30 nm	*n* = 10	ASTM D790-03 for FS and FM, ASTM D-638 for TS, Vicker's hardness test	Adding a mixture of HNTs and MWCNTs to PMMA may help to enhance the mechanical characteristics of PMMA denture bases, which may enhance their overall performance, wear resistance, and durability	[118]

Zirconia										

Khan et al. (2016)	Single walled/multiple walled/both	To assess the impact of SWCNTs and/or MWCNTs mixed with plain silanes and experimental silanes (ACPS + BTSE) on zirconia adhesion promotion to resin composite cement	SWCNT and MWCNT were oxidized (functionalized) by acid treatment with sulfuric and nitric acids (180 mL of H_2_SO_4_ and 120 mL of HNO_3_, respectively) at 50°C and refluxed for 24 h. The oxidized SWCNTs were then vacuum dried at 80°C for 12 h	Oxidized SWCNT and MWCNT were ultrasonically blended with 1 g of plain silane or 1 g of experimental silane primer containing 1.0 vol% of ACPS in 95.0 vol%/5.0 vol% ethanol/water, with a pH of 4.5 and a cross-linker silane 0.5 vol% BTSE	0.5 wt% of CNT in silane primer of all study groups	SWCNT: 9.5 nm, 1 = 1.5 nm ≥70% MWCNT: 9.5 nm, 1 = 1.5 nm ≥90%	*n* = 10	Atomic force microscope (AFM) analysis, surface wettability analysis, surface morphology and elemental analysis, enclosed mold shear bond strength (EM-SBS) using UTM, failure mode analysis using SEM	The adhesion strength (EM-SBS) of silica treated zirconia to resin composite cement was significantly increased by the infusion of oxidized SWCNTs blended with novel silane primer containing 1.0 vol% ACPS and 0.5 vol% BTSE	[119]

Jang et al. (2021)	Multiple-walled CNT	To investigate how MWCNTs affect the mechanical characteristics and microstructure of CNT/3YSZ composites by creating homogeneous samples with varying CNT contents using SPS	MWCNT particles were mixed with ethanol using 5 weight percent polyethylene imine and ultrasonicated for 1 h	MWCNTs were added to 3YSZ powder in various proportion to obtain homogenous composite powder which was subjected to multistage spark plasma sintering (SPS)	0, 1, 2, 3, 5, and 7 wt% of MWCNT slurry mixed with 3YSZ	20–50 nm	NA	Vickers indentation test, scanning electron microscopy (SEM), X-ray powder diffraction (XRPD)	7 wt% MWCNT/3YSZ composite is a desirable material for high-impact, high-temperature applications including environmental barrier coatings due to its high fracture toughness	[120]

Son et al. (2023)	Multiple walled CNT	To create 3D DRCs with various wt% of CNT and YSZ using the DLP method and assess their mechanical characteristics and stability after oral rinsing	Commercially available regular MWCNT	Various concentrations of CNT in wt% was mixed with pure resin by stirring rigourously at 1000 rpm for 2 h at 100°C by adding 1 wt% of dispersant	0.1, 0.3, 0.5, 0.7, 0.9 wt% of CNT	4.4 μm	NA	Rheology, laser-diffraction particle analysis, 3-point bending test, Rockwell hardness test, Oral rinsing stability, SEM study of fracture surface	Increased hardness, flexural strength, and reasonable oral rinse stability were found to be present in DRCs containing 0.5 wt% YSZ as compared to DRCs containing CNT	[121]

da Silva et al. (2023)	Multiple walled CNT	To evaluate the microstructure, flexural strength, fracture toughness and optical properties of the Y-TZP/MWCNT-SiO_2_ nanocomposite and compare these properties with those measured for conventional Y-TZP	Commercially available MWCNT	Functionalization of MWCNT with SiO_2_ surface coating by precursor hydroxide coprecipitation technique and hydrothermal treatment for coating the CNTs, followed by dispersion of the material in the commercial Y-TZP powder to obtain Y-TZP/MWCNT-SiO_2_ nanocomposite	NA	NA	NA	Microstructure analysis by SEM-FEG and TEM, TGA, DRX and FRX, Vickers hardness test, flexural strength, fracture toughness, optical properties	Y-TZP/MWCNT-SiO2 has satisfactory optical properties to be used in dental restorations but showed reduced flexural strength as compared to conventional Y-TZP which needs further improvements	[122]

Bone regeneration										

Zanello et al. (2006)	Single-walled (SW) CNTs and multiwalled (MW) CNTs	To examine which type of CNT—SWCNT or MWCNT is most effective in improving bone formation with various chemical modifications (electrically neutral, zwitterionic, or negatively or positively charged chemical groups)	SWNT-COOH, SWNT-PABS, SWNT-PEG based on their net negative, zwitterionic, and neutral electric charge, respectively, Sonication of in solvent (functionalized CNTs with water; AP-SWNTs and AP-MWNTs with 95% ethanol)	SWNT-COOH, SWNT-PABS, SWNT-PEG, AP-SWNTs, AP-MWNTs	NA	SWNTs:1.5 nm MWNT: 10–30 nm	NA	SEM- to study cell morphology and investigate the processes of bone production	Neutral charged- SWCNTs showed the highest cell growth with plate-shaped crystals formation, structurally similar to HA crystals in woven bone. Cells grown on neutral CNTs preserved osteoblast membrane electrical activity and improved Ca2+ channel functions, demonstrated a level of biocompatibility for AP-SWNTs and AP-MWNTs.	[123]

Gholami et al.	MWCNTs	To examine the mechanical strength and cytotoxicity of HAp/MWCNTs/bovine serum albumin (HA/MWCNTs/BSA) composites with different types of MWCNTs including hydroxylated and carboxylated MWCNTs (MWCNTs-OH, MWCNTs-COOH)	Commercially available MWCNT	(a) HA/MWCNTs/BSA (b) HA/MWCNTs-OH/BSA (c) HA/MWCNTs-COOH/BSA	6.25–200 μg/mL HA/MWCNTs/BSA	NA	*n* = 3 (mechanical Test)	Cell seeding and treatment of cells and MTT assay: for cytotoxic effect Mechanical test with instron 3367 universal testing machine: for mechanical testing	HA/MWCNTs-COOH/BSA showed the highest compressive strength. All developed composites at low concentration did not elicit cytotoxic effects on cell proliferation and HA/MWCNTs-COOH/BSA composites showed the highest viability. However, when the concentration was increased, it showed a reduction in cell viability	[124]

Tanaka et al. (2017)	MWCNT	To examine the mechanical strength and cytotoxicity of HAp/MWCNTs/bovine serum albumin (HA/MWCNTs/BSA) composites with different types of MWCNTs including hydroxylated and carboxylated MWCNTs (MWCNTs-OH, MWCNTs-COOH)	MWCNTs are synthesized by the chemical vapor deposition method and then underwent drying process in vacuo at 100°C for 24 h (carboxylated) MWCNT-COOH	rhBMP-2-containing MWCNT block	NA	20–40 nm	*n* = 5	Uniaxial compression tests-mechanical testing In vitro protein releasing assay Cell culture and seeding onto the scaffold Test for cell proliferation on MWCNT blocks Observation of cell adhesion by fluorescence microscopy Alkaline phosphatase activity assay	Ectopic bone was formed on the MWCNT blocks containing rhBMP-2 with the highest compressive strength similar to that of cortical bone. MWCNT blocks with rhBMP-2 were more stress resistant than MWCNT blocks without rhBMP-2	[125]

de Moura et al. (2020)	MWCNT	To examine the physical, biological and antimicrobial properties of PLA porous membranes and the synergistic effect with reinforcement of different concentrations of bioglass (BG) and CNTs GBR via in controlled humidity method	Chemical catalytic vapor deposition (CCVD)	PLA/BG/CNT	0.5, 1.0 and 1.5 wt%	9.5 nm	*n* = 15 (agar diffusion test)	Agar diffusion test: to evaluate antimicrobial activity MTT assay: Cell culture experiments to calculate he number of metabolically active cells in the porous membranes	The synergistic effect of BG and CNT on PLA membranes is more evident for PLA/5BG/1.0CNT which indicates excellent bioactivity, antimicrobial activity and excellent biocompatibility	[126]

Sanchez et al. (2021)	f-MWCNTs	To analyze the structure, mechanical, electrical properties and biocompatibility of chitosan-Hap-MWCNT (CS-HAp-MWCNT) films in different concentrations to develop a biocompatible flexible patch	Commercially available MWCNT	CS-HAp-MWCNT film	0.5 and 17 wt%	9.5 nm	SEM: >5 specimens . *n* = 18 (in vitro cell culture)	In vitro cell culture: cell viability SEM: The organization and the distribution of the HAp and MWCNTs into the CS matrix	Nanocomposite films with 5 wt% HAp and 0.5 wt% MWCNT demonstrated excellent mechanical strength with uniform distribution. CS-HAp-MWCNT film with 30 wt% HAp and 3 wt% MWCNT did not show uniform distribution due to the interaction between HAp and MWCNT leading to agglomeration of fillers (in >1 wt% MWCNT concentrations)	[127]

Silva et al. (2022)	MWCNT	To examine the effect of chitosan/CNTs scaffolds in addition to the application of the LLLT protocol on the regeneration of bone lesions	Commercially available MWCNT	G1: Control G2: Laser G3: C + CNTs, G4: C + CNTs + L, G5:C + CNTsM G6:C + CNTsM + L	0.25 mg of functionalized MWCN	9.5 nm	*n* = 30	SEM: The surface and calcium phosphate deposits EDX: to determine the ratio of Ca/P in the mineralized scaffold. Immunohistochemical analysis: To monitor the osteocytes development which is affected by osteocalcin (OC) and osteopontin (OPN)	Chitosan/CNTs scaffolds exhibit a homogenous porosity, and remains after the mineralization process. Bone neoformation was denser, thicker and voluminous in the scaffold of Mineralized Chitosan/CNTs with LLLT (C + CNTsM+L) with good biocompatibility	[128]

Tissue engineering										

Chen et al. (2013)	MWCNTs	To examine in situ precipitation as a new method to prepare chitosan–MWCNTs/hydroxyapatite(CS-MWNTs/ HAE) nanocomposite with good biocompatibility and high strength	Carboxylic MWCNT from Shenzhen Nanotech (Shenzhen, China)	CS-MWNTs/HA	Chitosan (Mw 1,000,000) with deacetylation of 95% degree	10–20 nm	NA	FTIR to investigate the electrostatic adsorption between MWCNTs and chitosan. Universal testing machine with stress–strain curve to evaluate mechanical properties. CCK-8 assay to evaluate cell proliferation on CS-MWNTs/HA nanocomposites	CS-MWNTs/HA nanocomposites showed increased elastic modulus and compressive strength and possess noncytotoxicity, which proves good biocompatibility. CSMWNTs/HA composites allow excellent attachment and adhesion of preosteoblast cells	[129]

Ignat et al. (2019)	Double-walled CNTs (DWCNTs)	To examine how the versatility of hASCs were affected during adipogenesis and osteogenesis by different concentrations of GO and CNTs reinforcement in a cellulose acetate (CA) membrane	Ammonia-functionalized short DWCNTs with over 90% carbon purity and 5% surface functionalization with NH_2_ groups	Cellulose acetate-CNT-graphene oxide CA-CNT-GO	CA-CNT-GO 0.25%, 0.5%, and 1%,	NA	*n* = 4 in micro-CT analysis.	Differentiation markers- gene and protein levels to be accessed Histological staining- to show how the processes evolve in response to CA-CNT-GO substrates. Micro-CT analysis: membrane dominant morphology	The total porosity and structural thickness of the CA membrane was minimum by addition of 1.0 wt% GO-CNT enrichment, with walls of increased thickness, enlarged and more homogenous pores which are important on cell behavior and improves structural integrity	[130]

Fathy et al. (2019)	f-CNTs	To assess how the composite of f-CNTs and chitosan (CS) influences the mechanical properties and cell biocompatibility of carbonated hydroxyapatite (HAp) derived from cuttlefish bone	f-CNTs (>8% functionalization)	HAp-CS-f-CNTs	Weight ratio of CS:f-CNTs of 2.5:1	9.5 nm	*n* = 30 20 cubic-shaped and 10 disc shaped HAp scaffold	Compressive strength test to evaluate the elastic modulus and the compressive strength FTIR- morphological assessments Cell proliferation assay- to evaluate cell viability	Compressive strength and elastic modulus (mechanical properties) were enhanced by HAp/CS/f-CNTs at low strain but the value was reduced in high stress due to brittle failure and progressive collapse of the microstructure in layers under compression. HAp group showed the highest mean in MSCs proliferating number and ALP (osteogenic ability) activity	[131]

Farshidfar et al. (2022)	MWCNT	To examine how the physical, chemical and biological properties of three-dimensional (3D) scaffold containing collagen (COL) were affected by different concentration of MWCNTs, and curcumin (CUR) reinforcement using freeze-drying technique	Functionalized MWCNTs	COL-MWCNTs 1%-CUR 10%	0.5%, 1%, and 1.5% *W*/*W* %	5–25 nm	3 grps: COL, COL-MWCNT, COL-MWCNT-CUR *n* = 30 (ultimate tensile strength test) *n* = 54 (in vivo biocompatibility)	Ultimate tensile strength test to evaluate stress–strain FTIR to detect chemical bonds between the materials and analyze their chemical components and composition. SEM and EDX to observe and confirm the hydroxyapatite (HAE) crystals on the surface of the scaffolds In vivo biocompatibility to evaluate the extent of inflammation, the reaction of multinucleated foreign body giant cells (FBGCs), and the level of vascularization	The COL-MWCNTs 1%-CUR 10% scaffolds revealed in vitro bioactivity, and in vitro biocompatibility with reduced inflammatory response in the rat animal model. The developed COL-MWCNTs 1%-CUR 10% composite scaffolds exhibited excellent surface wettability and significant decreased porosity	[132]

Periodontal treatment										

Meiet al. (2007)	MWCNTs	To examine how the biological characteristic of poly(L-lactic acid) Electrospun Membrane is affected by addition of MWCNTs/hydroxyapatite NPs (MWNTs/HAE) to develop a new type of guided tissue regeneration (GTR) membrane	MWNTs with purity >95%	PLLA/MWNTs/HA membrane	3 wt% MWNTs	20–40 nm	Nil	SEM and EDX analysis to evaluate the structure and presence of P and Ca elements in membranes Human PDLCs and GECs Culture on Membranes to examine cell adhesion and proliferation by cell counting and MTT assay In vivo implantation of PLLA/MWNTs/HA membranes with PDLCs: To perform histological analyses, including staining with hematoxylin and eosin, staining for calcium deposits using alizarin red, and immunohistochemical staining for osteocalcin (OC)	PLLA/MWNTs/HA is a single-layered material with dual biological functions, which enhances the adhesion and growth of human periodontal ligament cells (PDLCs) while suppressing the adhesion and growth of gingival epithelial cells (GECs). Selectivity of PLLA/MWNTs/HA membrane is demonstrated by its ability to increase the adhesion and growth of periodontal ligament cells (PDLCs) by 30% while reducing the adhesion and proliferation of gingival epithelial cells (GECs) by 30%	[133]

Suo et al. (2022)	Nil	To examine how the osteogenic properties of composite scaffold was affected by different concentration of graphene oxide/hyaluronic acid/chitosan (GO/HA/CS) reinforcement using 3D printing technique	Nil	Graphene oxide/hyaluronic acid/chitosan (GO/ HA/CS) composite hydrogel	0.1%, 0.25%, 0.5%, 1% GO/HA/CS	Nil	Nil	SEM to observe the surface morphology of the composite scaffold Physical and chemical properties to test its mechanical properties, water swelling rate, in vitro degradation and other material properties. Biological evaluation focused on cell morphology, adhesion, proliferation, and live-dead staining	Multilayered GO/HA/CS biological scaffold was constructed with an interconnected microporous structure. Appropriate amount of GO such as 0.25% has very minimal cytotoxicity and enhances cell growth with excellent biocompatibility with osteoblasts which helps in repair of bone defect	[134]

Zarei et al. (2020)	MWCNTs	To examine how the physical, chemical and biological properties of Poly (3-hydroxybutyrate) electrospun scaffold is affected by incorporation of 1% CNTs for periodontal regeneration	Functionalized multiwalled carbon nano-tubes (COOH) with purity >95 wt%	PHB/1%CNTs	1 wt% of functionalized CNTs	5‒25 nm	*n* = 25 (in vitro human PDLSCs culture) *n* = 5 (SEM images) *n* = 5 (bioactivity evaluation) *n* = 5 (animal study)	SEM and universal testing machine to evaluate the physical properties of the scaffolds. FTIR: to evaluate the chemical characterization of the scaffolds Biological properties of the scaffolds were also evaluated in vitro by culturing periodontal liga- ment stem cells (PDLSCs) on the scaffolds for 10 days and in vivo by Implanting the scaffolds in a rat model for 5 weeks	The mechanical properties and tensile strength of PHB/1%CNTs was enhanced, similar to human PDL. PHB/1%CNTs formed a scaffold similar to collagen fibrous structure in the PDL, with increased fiber diameter and porosity. Functionalized CNTs improved the wettability, bioactivity, in vitro and in vivo biocompatibility	[135]

Endodontic treatment										

Sanaee et al. (2019)	MWCNT	To examine the effect of particle size distribution and physical properties of mineral trioxide aggregate (MTA) with reinforcement of different concentrations of MWCNT	Commercially available MWCNT	MTA-MWCNT	0.25, 0.5, 0.75 and 1 wt%	Outer diameter: 10–20 nm Inner diameter: 5–10 nm	*n* = 5 (particle size analysis) *n* = 15 (compressive and flexural strength)	SEM: Observe the surface of MTA flexural and compressive strength Photographic densitometer: radiopacity	The flexural strength and compressive strength were increased in 0.25 wt%, 0.50 wt% and 0.75 wt% MWCNTs reinforced MTA specimens but reduced in 1.0 wt% MWCNTs-MTA	[136]

Baghdadi et al. (2020)	Multiwalled carbon nanotubes	To examine how physiochemical properties of BioRoot root canal sealer is affected by reinforcement of different nanomaterial concentration: MWCNTS, titanium carbide (TC) or boron nitride (BN; 1 and 2 wt%)	NA	Bioroot/1 wt%BN Bioroot/1 wt% CN BioRoot/1 wt% TC	1 and 2-wt%	3–5 nm (internal); 6–13 nm (external)	*n* = 3	Setting time Solubility: difference between the initial mass of the Petri dish and its final mass Elution: the difference between the mass of the specimen before and after it has been immersed in water. pH Measurement SEM: to examine the microstructure of the composite materials. Energy dispersive microanalysis detector: for elemental analysis	The initial and final setting times of 1 wt% composites were significantly shorter than BioRoot RCS and 2 wt% composites. Bioroot/1 wt%BN had the shortest initial setting time but Bioroot/1 wt% CN had the shortest final setting time	[137]

Baghdadi et al. (2021)	MWCNTs	To examine the microstructural properties and compressive strength of BioRoot RCS with reinforcement of different concentrations of MWCNTs, titanium carbides (TC) and boron nitrides (BN)	NA	Bioroot/1-wt% CN Bioroot/2-wt% CN Bioroot/1-wt% TC Bioroot/2-wt% TC Bioroot/1-wt% BN Bioroot/2-wt% BN	1 and 2 wt%	3–5 nm (internal); 6–13 nm (external)	*n* = 70	X-ray diffraction (XRD): used to examine the phase structure of the unmodified BioRoot RCS and the resulting composites. FTIR: to identify functional groups, assess the formation of intermolecular bonds, and analyze the distribution of various NPs compared to the unmodified BioRoot RCS. Compressive strength test: SEM: investigate microstructural properties such as microcracks	The 1 wt% Bioroot/MWCNTs and Bioroot/TC showed a significant improvement in the compressive strength, whereas 2 wt% composites did not show any increase. As the concentration of MWCNT and TC increases, progression of microcracks and fracture of the NPs appeared along the surface indicating a brittle mode of fracture	[138]

Marica et al. (2021)	Multi-walled CNTs	To examine the physico-chemical, structural and morphological characterization, nanomechanical properties, antibacterial and antifungal properties of a nanomodified endodontic sealer, with reinforcement of MWCNTs, encapsulating chlorhexidine (CHX) and colloidal silver NPs (AgNPs)	Commercially available MWCNT	CNTs/AgNPs/CXH 2%; CNTs/H2O; CNTs/AgNPs; CNTs/CXH 2%; AgNPs; CHX 2%	12.5 mg CNTs	9.5 nm	*n* = 20	FTIR spectroscopy: to assess the structural features of both standard and nanomodified endodontic sealers EDX analysis: to do the microstructure investigations Electrochemical analysis: to assess the electrochemical behavior of the colloidal mixture and of its individual components, differential pulse voltammetry (DPV) SEM: to evaluate each individual specimen in terms of interfacial adaptation toward modified and unmodified sealer. Nanoindentation measurements and thermogravimetrical analysis (TGA) antimicrobial and antifungal efficacy	Reinforcement of CNTs/CHX/AgNPs in the epoxy-based sealer improved the nanomechanical properties of the composite. CNTs/AgNPs/CHX 2% mixture demonstrated best efficacy against E.faecalis. the addition of CNT to AgNP or CHX significant enhanced the antimicrobial effect. Modified sealer incorporating CNTs, CHX2% and AgNPs provided improved antibacterial effect without compromising structural integrity and thermal properties of the sealer	[139]

Cancer treatment										

Das et al. (2023)	MWCNT and SWCNT	To study the recent development of carbon-based nanomaterials to aid in cancer diagnosis	NA	NA	NA	NA	NA	NA	SWCNTs exhibit outstanding sensitivity, photostability, and near-infrared (NIR) photoluminescence, enabling them to perform real-time optical measurements of hybridization of microRNA and other oligonucleotides	[140]

Li et al. (2023)	NA	To evaluate the CNT-modified nanodrug delivery system (CNTs/Gel/Cp) for their drug-loading capacity, encapsulation effectiveness, and release properties and anticancer effect in vitro	Commercially available CNT	CNTs/Gel/Cp	Surface modification of 0.25 g of CNTs with PDA, then addition of 0.5 g of gelatin. Then, 10 mg CNTs/Gel added with 10 mg of cisplatin	NA	*n* = 3 (in vitro release studies)	SEM: microscopic morphology of the samples in CNTs/Gel and CNTs/Gel/Cp TEM and EDS: to identify the types, amount, and distribution of the chemical elements. Dynamic light scattering (DLS): to measure the average diameter and size distribution of the CNTs/Gel and CNTs/Gel/Cp. XRD spectra and Raman spectroscopy: to identify crystal phases and analyze the structural characteristics of solid materials. In vitro antitumor effect: to determine the viability of human tongue squamous cell carcinoma cell Cal-27	CNTs/Gel/Cp demonstrated a homogenous distribution of nanotubes with rougher surface which enhanced its adsorption to cisplatin loading. The ratio of MCp:MCNTs/Gel = 1:1 showed the maximum loading rate and entrapment efficiency with effective sustained release in a pH 6.0 environment	[141]

Lu et al. (2021)	SWCNT and MWCNT	To review the recent advancements in CNT–based drug delivery systems for cancer theranostics, focusing on their ability to target both intracellular and extracellular components of tumor cells and its clinical application in diagnosis	NA	NA	NA	NA	NA	NA	Functionalized CNTs are effective carriers for both biomolecules and small molecule anticancer drugs, providing improved biocompatibility, biodegradability, and targeted delivery. This allows the drugs to be precisely delivered to their target sites with significantly lower drug concentrations, achieving more effective outcomes compared to drugs administered in their free form	[142]

Chary et al. (2023)	SWCNT and MWCNT	To review on the application of theranostic multifunctional carbon nanotubes (mf-CNTs) as a carrier for potent treatments in diagnosing and treating different cancers to enhance the safety and effectiveness of chemotherapy	NA	NA	NA	NA	NA	NA	The mf-CNTs serve multiple functions in pharmaceuticals, such as imaging techniques for cancer detection, photothermal and photodynamic therapies and as nanocarriers for delivering anticancer drugs, genetic materials, and proteins	[143]

Sowmya et al. (2023)	NA	Use of NP-based biosensors in detecting biomarkers in oral squamous cell carcinoma (OSCC)	NA	NA	NA	NA	NA	NA	NP-based biosensing offers high sensitivity and specificity for early cancer detection, and this aids clinicians in accurate diagnosis, prognosis, and targeted drug delivery while minimizing adverse effects. Besides, it can detect multiple biomarkers more accurately, track tumor cells and deliver drugs to the target sites precisely	[144]

Dental implants										

Esmaili et al. (2022)	MWCNTs	Aimed to improve the corrosion resistance of the Ti-6Al-4V implant and decrease the release of harmful ions from the coated Ti alloy by using tantalum carbide (Ta_2_C) / MWCNTs multilayer thin film of was deposited on the Ti-6Al-4 V	Electrophoretic technique	Ta_2_C/MWCNTs multilayer thin film of was deposited on the Ti-6Al-4 V	95%	>50 nm	NA	Energy-dispersive X-ray analysis (EDX), potentiostat/galvanostat, nanomechanical instrument, methyl thiazolyl tetrazolium (MTT)	Ta_2_C/MWCNT coating layer improved the fracture toughness, tribological properties, and the resistance to plastic deformation	[145]

Malekahmadi et al. (2021)	MWCNTs	Aimed to find optimal combination and temperature of synthesized hydroxyapatite CNT-water hybrid nanofluid achieving both the biocompatibility and the thermal conductivity for dental implant coating	Dispersion	MWCNT was added to the mono-nanofluid of HA (0.2, 0.4, 0.6, 0.8, and 1.0 vol%), by the volumetric ratio of 1:1 for HA/CNT to obtain a hybrid nanofluid (HN)	NA	<100 nm	NA	X-ray powder diffraction (XRD), FTIR, Raman spectroscopy, field emission scanning electron microscope (FESEM), and energy-dispersive X-ray spectroscopy (EDS)	Highest enhancement of thermal conductivity is 25.83% for the 1.0 vol% composite sample of CNT and temperature of 50°C compared with mono nanofluids	[146]

Sivaraj D et al. (2020)	MWCNTs	Investigated the potential change in the antibacterial properties of Cu-hydroxyapatite/ functionalized MWCNT (HAE/f-MWCNT) composite coated heterogeneous implant surfaces against gram positive and gram-negative microorganism and potential effect on surface corrosion effects occurring in stimulated body fluids	Spray pyrolysis deposition technique	HA/f-MWCNT	5–25 mg/ml	<100 nm	NA	XRD, FTIR, FESEM, and EDS	When compared to HA/f-MWCNT, the hybrid Cu-HA-MWCNT composite demonstrated superior antibacterial activity against gram-positive and gram-negative bacteria, with a maximal inhibition zone of 13–17 mm. When compared to other microorganisms, the Cu-HA/f-MWCNT nanocomposites demonstrated good antibacterial activity against *E. coli*	[147]

## Nomenclature

BC: Bacterial cellulose
BN: Boron nitride
CNPs: Carbon nanoparticles
CS-MWNTs/HAE: Carbon nanotubes/hydroxyapatite
CNT/Gel/Cp: Carbon nanotube (CNT)-modified nano-drug delivery system
CA: Cellulose acetate
CVD: Chemical vapor deposition
CHX: Chlorhexidine
Cp: Cisplatin
COL: Collagen
AgNPs: Colloidal silver nanoparticles
CUR: Curcumin
CNT/CS/AL: CNT/chitosan/sodium alginate
DWCNTs: Double-walled CNTs
DOX: Doxorubicin
FTIR: Fourier transform infrared spectroscopy
f-CNTs: Functionalized carbon nanotubes
GEC: Gingival epithelial cell
Au NC/MWCNT–NH_2_: Gold nanocage and amidated multiwalled carbon nanotube
GBR: Guided bone regeneration
GTR: Guided tissue regeneration
GNPs: Graphene nanoplatelets
GO/HA/CS: Graphene oxide/hyaluronic acid/chitosan
GQDs: Graphene quantum dots
rGO-MWCNT/AuNP: Graphene oxide-multiwalled carbon nanotube platform with gold nanoparticles
HA NP: Hydoxyapatite nanoparticles
HAE/HDPE: Hydroxyapatite, high density polyethylene
HepG2: Human adipose-derived stem cells (hASCs), human liver cancer
LSPR: Localized surface plasmon resonance
MSCs: Mesenchymal stem cells
MTA: Mineral trioxide aggregate
MWCNTs: Multiwalled carbon nanotubes
mf-CNTs: Multifunctional carbon nanotubes
NPs: Nanoparticles
OSCC: Oral squamous cell carcinoma
PDL: Periodontal ligament
PDLC: Periodontal ligament cell
uPA: Plasminogen activator
PMMA: Polymethyl methacrylate
PLLA: Poly(L-lactic acid)
PHB: Poly(3-hydroxybutyrate)
PEGylated SWNTs: Polyethylene glycol single-wall carbon nanotubes
PSA: Prostate-specific antigen
ROS: Reactive oxygen species
SEM: Scanning electron microscope
SWCNTs: Single-walled carbon nanotubes
SPR: Surface plasmon resonance
TC: Titanium carbide
TiNbZr: Titanium–niobium–zirconium
TEM: Transmission electron microscopy
TME: Tumor microenvironment
WCA: Water contact angle.
